# Genome-Wide Identification, Expression Patterns and Sugar Transport of the Physic Nut *SWEET* Gene Family and a Functional Analysis of *JcSWEET16* in *Arabidopsis*

**DOI:** 10.3390/ijms23105391

**Published:** 2022-05-12

**Authors:** Youting Wu, Pingzhi Wu, Shaoming Xu, Yaping Chen, Meiru Li, Guojiang Wu, Huawu Jiang

**Affiliations:** 1 Key Laboratory of South China Agricultural Plant Molecular Analysis and Genetic Improvement, Provincial Key Laboratory of Applied Botany, South China Botanical Garden, Chinese Academy of Sciences, Guangzhou 510640, China; wuyouting@scbg.ac.cn (Y.W.); mingming_er@126.com (S.X.); chenyp@scbg.ac.cn (Y.C.); limr@scbg.ac.cn (M.L.); wugj@scbg.ac.cn (G.W.); 2 University of Chinese Academy of Sciences, Beijing 100049, China; 3 Key Laboratory of South Subtropical Fruit Biology and Genetic Resource Utilization, Ministry of Agriculture/Key Laboratory of Tropical and Subtropical Fruit Tree Research of Guangdong Province, Institution of Fruit Tree Research, Guangdong Academy of Agricultural Sciences, Guangzhou 510640, China; pzwu@scbg.ac.cn

**Keywords:** *SWEET* gene family, sugar transporter, gene evolution, gene expression, abiotic stress, physic nut

## Abstract

The Sugars Will Eventually be Exported Transporters (SWEET) family is a class of sugar transporters that play key roles in phloem loading, seed filling, pollen development and the stress response in plants. Here, a total of 18 *JcSWEET* genes were identified in physic nut (*Jatropha curcas* L.) and classified into four clades by phylogenetic analysis. These *JcSWEET* genes share similar gene structures, and alternative splicing of messenger RNAs was observed for five of the *JcSWEET* genes. Three (*JcSWEET1*/*4*/*5*) of the JcSWEETs were found to possess transport activity for hexose molecules in yeast. Real-time quantitative PCR analysis of *JcSWEETs* in different tissues under normal growth conditions and abiotic stresses revealed that most are tissue-specifically expressed, and 12 *JcSWEETs* responded to either drought or salinity. The *JcSWEET16* gene responded to drought and salinity stress in leaves, and the protein it encodes is localized in both the plasma membrane and the vacuolar membrane. The overexpression of *JcSWEET16* in *Arabidopsis thaliana* modified the flowering time and saline tolerance levels but not the drought tolerance of the transgenic plants. Together, these results provide insights into the characteristics of *SWEET* genes in physic nut and could serve as a basis for cloning and further functional analysis of these genes.

## 1. Introduction

Sugars are the predominant product of plant photosynthesis; they not only participate in the storage and transportation of nutrients but also play important roles in signal transduction and stress responses [1,2,3]. Sugars are transported from the source tissue to the sink tissue through phloem in order to maintain normal plant growth and development. However, sugars cannot be transported independently across plant membranes; therefore, their transmembrane movement requires sugar transporters [4,5,6]. Currently, three families of transporters involved in intercellular sugar transport have been identified, including MSTs (monosaccharide transporters), SUTs (sucrose transporters) and SWEETs (Sugars Will Eventually be Exported Transporters) [7]. MSTs and SUTs contain 12 transmembrane domains (TMs) that belong to the major facilitator superfamily. However, SWEETs are structurally a different type of transporter, with seven TMs, and are classified with the MtN3/saliva family (PF03083) of transmembrane transporters [4]. Phylogenetic analysis has demonstrated that plant *SWEET* family genes can be divided into four clades. Different family members have distinct patterns of tissue expression and participate in the transport of diverse sugar molecules. The subcellular localization of family members also varies, indicating that *SWEET* family genes have numerous biological functions that are extremely important for plant growth and development [8,9,10].

The number of *SWEET* genes varies across species, and the number present within a species does not correlate with the evolutionary complexity of that species; plants have larger numbers of *SWEET* gene family members than do other organisms [11]. To date, *SWEET* genes have been identified in a number of plants, including *A. thaliana* (*Arabidopsis thaliana*), rice (*Oryza sativa*), soybean (*Glycine max*) and maize (*Zea mays*). AtSWEET1 was the first plant SWEET protein to be identified; it transports glucose and is highly expressed during early flower development [4,12]. Chen et al. [13] found that AtSWEET11 and AtSWEET12 were localized to the plasma membrane of phloem parenchyma cells and are involved in sucrose transport. The double mutation of *AtSWEET11* and *AtSWEET12* affected sucrose phloem transport, indicating for the first time the key role of these plant SWEET proteins in phloem loading [14,15]. *AtSWEET11*, *AtSWEET12* and *AtSWEET15* are expressed during seed development, especially in maternal tissues; the triple mutant of *atsweet11;12;15* showed severe seed defects, indicating that SWEET proteins are involved in the seed filling process in *A. thaliana* [16]. *AtSWEET8* (Ruptured Pollen Grain 1, RPG1) was found to be strongly expressed in the microsporocyte (or microspores) and tapetum during male meiosis, and the *rpg1* mutant exhibited severely reduced male fertility [17]. AtSWEET9 is a nectary-specific sugar transporter in this eudicot species and is essential for nectar production [18]. Recently, studies have shown that SWEET proteins are also involved in plant hormone transport. AtSWEET13 and AtSWEET14 participate in the transport of different gibberellins (GAs) and regulate GA-mediated physiological processes, including anther dehiscence and seed development [19]. *OsSWEET11* and *OsSWEET15* also play an important role in rice seed filling [20,21,22]. In soybean, most of the SWEET genes are expressed in seeds, and the mutation of *GmSWEET15* results in retarded seed embryo development [23,24]. In maize, *ZmSWEET13* paralogues (a, b, c) are among the most highly expressed genes in the leaf vasculature, and a triple mutant of the three *ZmSWEET13* paralogues exhibited impaired phloem loading [25].

Soluble sugars are major osmolytes, and SWEET proteins can participate in plant stress responses by regulating the allocation of soluble sugars. In *A. thaliana*, *AtSWEET16* and *AtSWEET17* are involved in abiotic stress responses, and further, the overexpression of *AtSWEET16* can increase the tolerance of cold stress [26,27]. *AtSWEET15* (SAG29) is expressed primarily in senescing plant tissues; SAG29-overexpressing transgenic plants were hypersensitive to salinity stress, and the SAG29-deficient mutants were less sensitive to high salt levels [28]. The expression of *AtSWEET11* and *AtSWEET12* were down-regulated by cold treatment, and the double mutation of *AtSWEET11* and *AtSWEET12* exhibited greater freezing tolerance than the wild-type and both single mutants [29]. AtSWEET4 is a hexose facilitator, and the overexpression of *AtSWEET4* in *A. thaliana* increased plant size and exhibited higher freezing tolerance [30]. In rice, OsSWEET13 and OsSWEET15 are major SWEET transporters regulating both sucrose transport and levels in response to drought and salinity stresses by the binding of an ABA-responsive transcription factor OsbZIP72 to the promoters [31]. CsSWEET2 is a hexose transporter from Cucumber (*Cucumis sativus*), and it plays a vital role in improving plant cold tolerance by mediating sugar metabolism and compartmentation [32]. A total of 22 *ClaSWEET* genes were identified in the watermelon (*Citrullus lanatus*) genome, and the expression patterns of *ClaSWEET* genes demonstrated that ClaSWEET proteins play key roles in responses to abiotic stresses, including drought, salt levels and low-temperature stresses [33]. In *Camellia sinensis*, CsSWEET16 contributes to sugar compartmentation across the vacuole and functions in modifying cold tolerance when overexpressed in *A. thaliana* [34].

Physic nut is a small perennial tree or large shrub of the Euphorbiaceae family; it has attracted wide attention due to its fast growth, ease of propagation, tolerance of poor-quality land, considerable adaptability and the high oil content of its seeds [35]. Genome sequence and expression sequence tag (EST) libraries constructed by our team and others in recent years provide a solid basis for the analysis of physic nut gene families and their evolution [36,37,38]. In this study, a genome-wide analysis was conducted to identify the *SWEET* genes of physic nut. We analyzed the exon-intron structure and the phylogenetic relationships of *JcSWEET* genes in detail and examined the expression levels of *JcSWEET* genes in different tissues under normal growth conditions and abiotic stresses. The cDNA clones of 13 JcSWEETs were obtained and transferred to yeast to test sugar transport activities. Further study of the function of *JcSWEET16* revealed that it is localized in the plasma membrane and vacuolar membrane and has roles in flowering time and saline tolerance when overexpressed in *A. thaliana*.

## 2. Results

### 2.1. Identification and Phylogenetic Analysis of SWEET Family Genes in Physic Nut

Based on the domain sequences of *A. thaliana* and rice proteins, a total of 18 putative *JcSWEET* genes were identified from the published genome database [36,37] using a BLAST search analysis approach. The lengths of the coding sequences of *JcSWEET* genes ranged from 708 bp to 918 bp, and they encoded polypeptides containing 235 (JcSWEET2a/2b) to 305 (JcSWEET16) amino acids (Table S1). These *JcSWEET* genes were named based on their homologs in *A. thaliana*. All SWEET proteins were predicted to have seven transmembrane domains (TMs) (Figure S1). There are approximately 86 to 91 amino acids in the two MtN3/saliva domains, and their positions in all proteins are similar. Multiple sequence alignments of the 18 JcSWEETs and AtSWEET1 (AT1G21460) proteins revealed that all TMs were relatively conserved except for the fourth, which is characteristic of SWEET proteins (Figure S2).

**Figure 1:**
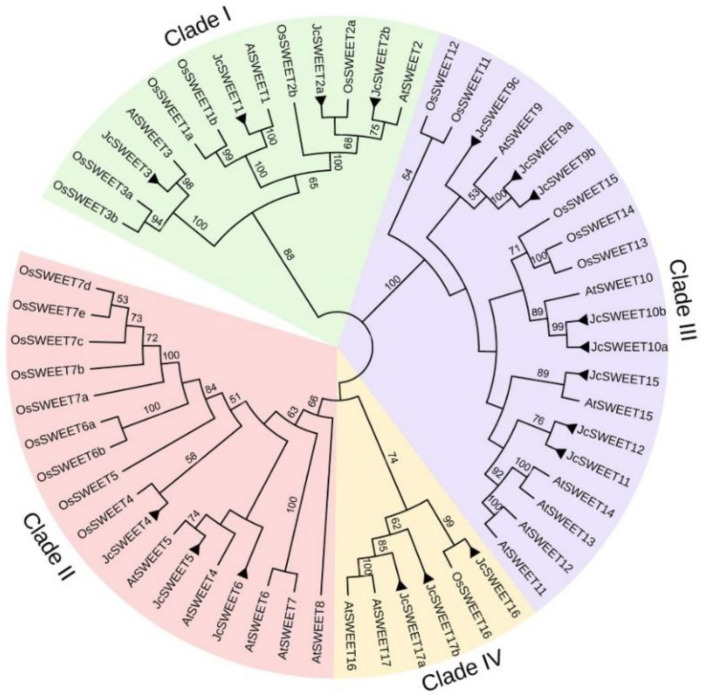
Phylogenetic relationships of *SWEET* family genes in physic nut, rice, and *A. thaliana*. The sequences of the SWEET proteins from the above three plant species were aligned by CLUSTAL_X, and the phylogenetic tree was constructed using MEGA 5.0 and the neighbor-joining (NJ) method with default settings. Jc, *Jatropha curcas*; At, *A. thaliana*; Os, *Oryza sativa*. *JcSWEET* genes are identified by black triangles.

To investigate the phylogenetic relationships among the *SWEET* genes in physic nut and other plant species, a phylogenetic tree was constructed by aligning 18 JcSWEET protein sequences, 17 AtSWEET protein sequences from *A. thaliana* and 21 OsSWEET protein sequences from rice using the program MEGA5.0. According to this phylogenetic tree, all JcSWEET proteins could be clustered into four clades, as previously reported for *A. thaliana* [4]. Clade I (JcSWEET1/2a/2b/3) contains four JcSWEET proteins, clade II (JcSWEET4/5/6) and clade IV (JcSWEET16/17a/17b) both contain three JcSWEET proteins, and the remaining eight JcSWEET proteins all belong to clade III (JcSWEET9a/9b/9c/10a/10b/11/12/15) (Figure 1).

### 2.2. Gene Structure and Chromosomal Distribution Analysis of SWEET Family Genes in Physic Nut

**Figure 2:**
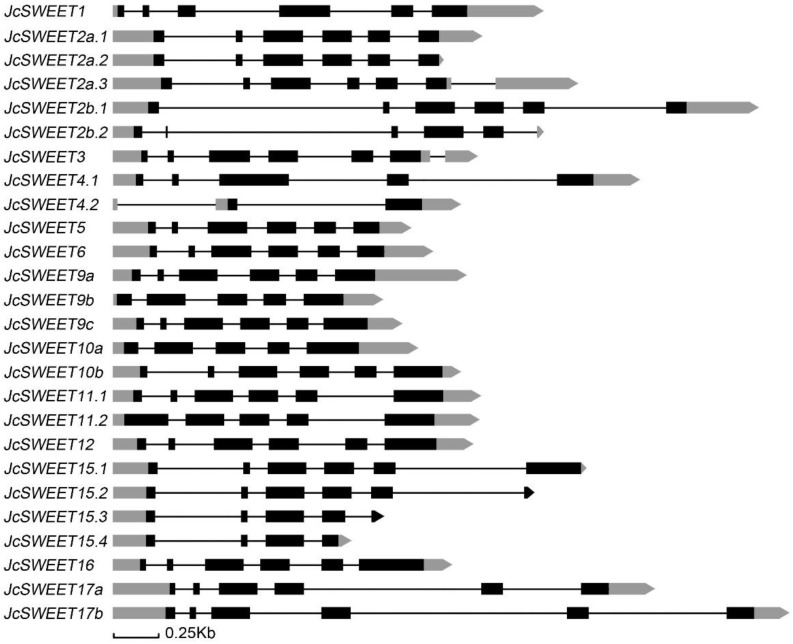
Structures of the 18 *JcSWEET* genes. The exons are shown as boxes (coding sequence (CDS) in black, untranslated region (UTR) in gray), while the introns are represented by lines.

To analyze the structural characteristics of *JcSWEET* genes, we mapped their structures by submitting their full-length coding sequences and the corresponding genomic DNA sequences to the online Gene Structure Display Server (http://gsds.gao-lab.org/ (accessed on 19 January 2022)). All *JcSWEET* genes shared a similar exon-intron arrangement, with four to five introns in the coding region. The *JcSWEET4* gene did not contain the third intron of other *JcSWEET* gene family members, while *JcSWEET9b* and *JcSWEET10a* did not contain the first intron of other family members. Alternative splicing of messenger RNAs was observed for five *JcSWEET* genes, including family members *JcSWEET2a*, *JcSWEET2b*, *JcSWEET4*, *JcSWEET11* and *JcSWEET15* (Figure 2).

Via the use of our previously constructed linkage map [36], 16 of the 18 *JcSWEET* genes were mapped to 8 of the 11 linkage groups (LGs), but the two remaining family members (*JcSWEET1* and *JcSWEET2b*) were located on unmapped scaffolds. The genes were unevenly distributed on LGs, and no *JcSWEET* gene was found on LGs 2, 10 or 11. The highest concentration was located on LG 5, which contained six genes (*JcSWEET3*/*5*/*10a*/*10b*/*11*/*12*). Tandem duplication, defined as tandem repeats that are located within 50 kilobases (kb) of each other or are separated by <4 non-homologous spacer genes [39], was observed for *SWEET* genes in the physic nut genome. Tandem duplications were observed on LG 5, which contains four genes (*JcSWEET10a*, *10b*, *11* and *12*) that were grouped into the same clade (Clade III) of the phylogenetic tree (Figure S3).

In order to test whether these tandem duplicates arose from recent duplication events in physic nut, we constructed another phylogenetic tree using SWEET proteins from physic nut and a closely related species, the castor bean (*Ricinus communis*) (Figure S4). Based on this phylogenetic tree, these four tandem duplications are also present in the castor bean genome. Paralogs of *JcSWEET10a*/*10b* and *JcSWEET11*/*12* were also observed as a tandem pair in the genomes of *A. thaliana* (*AT5G50790*/*AtSWEET10*-*AT5G50800*/*AtSWEET13*). These results indicate that these tandem repeats in physic nut have resulted from both ancient (in Dicotyledoneae) and recent (in Euphorbiaceae) gene duplication events.

### 2.3. Expression Profiles of SWEET Genes in Different Physic Nut Tissues

**Figure 3:**
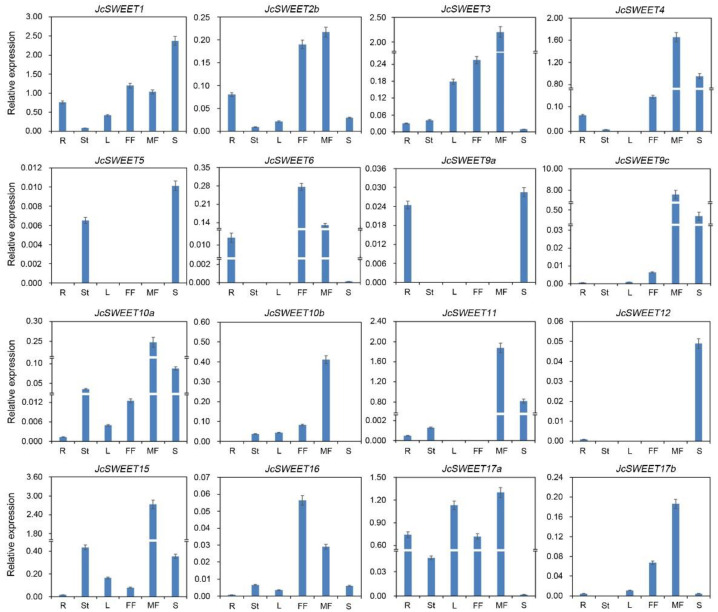
Expression analysis of *JcSWEET* genes in physic nut plants under normal conditions. R, root; St, stem cortex; L, leaf; FF, female flower; MF, male flower; S, seed. Relative expression was normalized to that of the reference gene *JcActin* (internal control).

To study the expression patterns of *JcSWEET* genes and gain information about their roles in the growth and development of physic nut, quantitative RT-PCR (qRT-PCR) was performed to measure transcription levels in various tissues and organs, including the roots, stem cortex, leaves, female flowers, male flowers and developing seeds. Although the expression of almost all genes could be detected in the tissues tested, except for family members *JcSWEET2a* and *JcSWEET9b*, the expression of each family member varied greatly (Figure 3). Genes within the same clade also displayed considerable differences in their level of expression across the different tissues analyzed.

**Figure 4:**
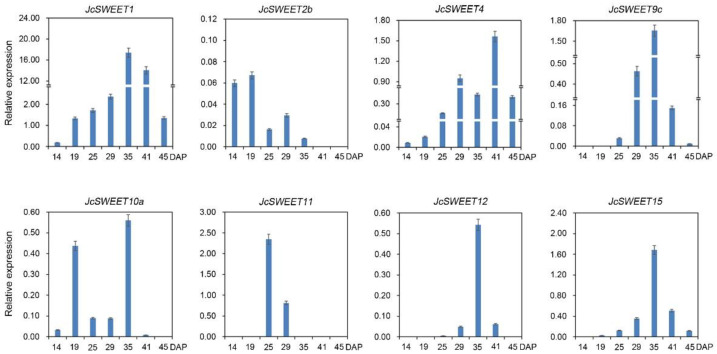
Patterns of expression of *JcSWEET* genes of seeds from 14 to 45 days after pollination (DAP). The relative expression was normalized to that of the reference gene *JcActin* (internal control).

In clade I, *JcSWEET1* was highly expressed in developing seeds (Figure 3), and its expression level was consistent with the seed filling process (Figure 4). *JcSWEET2b* was expressed in early developing seeds at low levels (Figure 4), while *JcSWEET3* was expressed at a low level in developing seeds but highly in male flowers (Figure 3). In clade II, *JcSWEET4* was expressed mainly in roots, flowers and seeds. *JcSWEET5* was weakly expressed in all the tissues tested, while *JcSWEET6* was expressed at a relatively higher level in flowers (Figure 3). In clade III, only a low level of expression was detected for *JcSWEET9a* across the assessed tissues, while a relatively high level of expression for *JcSWEET9c* was detected in flowers and seeds at the middle stage of development (Figure 3 and Figure 4). *JcSWEET15* was highly expressed in the stem cortex, male flower, and filling-stage seeds. For the tandem duplication *JcSWEET10a*/*JcSWEET10b*, transcripts of the *JcSWEET10a* gene were detected in seeds at the early and middle developmental stages, whereas only a low level of expression was observed for *JcSWEET10b* in developing seeds. Both *JcSWEET11* and *JcSWEET12* were expressed at low levels in roots, leaves, and the stem cortex, but these two family members were relatively highly expressed in seeds at the middle developmental stage (Figure 4). In addition, *JcSWEET10a* and *JcSWEET11* were highly expressed in male flowers (Figure 3). In clade IV, the relative expression level of *JcSWEET17a* was higher than those of the others in the tested tissues under normal growth conditions.

### 2.4. Expression Profiles of JcSWEET Genes under Drought and Salinity Stress

**Figure 5:**
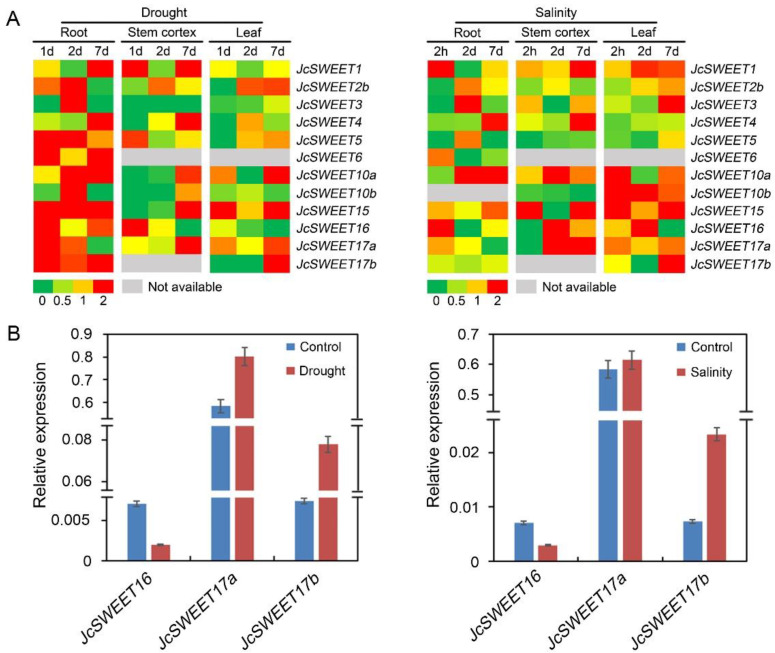
Expression patterns of *JcSWEET* genes in response to drought and salinity stresses. (**A**) Heatmap showing the expression levels of *JcSWEET* genes under drought and salinity stresses. Values presented in the heatmap are the ratios of stress treatment to control. (**B**) Expression levels of selected *JcSWEET* genes in leaves under drought and salinity stress at the 7 day point measured using qRT-PCR. Relative expression was normalized to that of the reference gene *JcActin* (internal control).

To determine the roles of *JcSWEET* genes in abiotic stress responses, the expression patterns of *JcSWEETs* under drought and salinity stress were analyzed using our next-generation sequencing-based digital gene expression tag database [40,41]. Overall, a total of 12 out of the 18 *JcSWEET* genes showed differential expression levels in response to at least one stress in at least one tissue. *JcSWEET3*/*10a* exhibited similar expression patterns under drought and salinity stress and were significantly up-regulated in roots after 2 days of treatment, and the expression level of *JcSWEET17b* also increased markedly in leaves after 7 days of exposure to both imposed stresses. The expression level of *JcSWEET15* increased dramatically in roots under drought stress but showed no significant response to salinity stress. The level of *JcSWEET16* transcript increased in leaves but decreased in roots after salinity treatment for 2 days (Figure 5A).

To verify the expression profiles determined using the digital gene expression tag database, qRT-PCR was employed to analyze the expression levels of clade IV (*JcSWEET16*/*17a*/*17b*) genes in leaves under drought and salinity stresses at the day 7 time point. This analysis showed that *JcSWEET17a*/*17b* were up-regulated and *JcSWEET16* was significantly down-regulated in leaves under drought and salinity stress treatments (Figure 5B); these findings were consistent with the corresponding transcript abundance changes obtained by Digital Gene Expression Profiling, indicating that the digital expression data were reliable.

### 2.5. Transport Activity of JcSWEETs in Yeast

**Figure 6:**
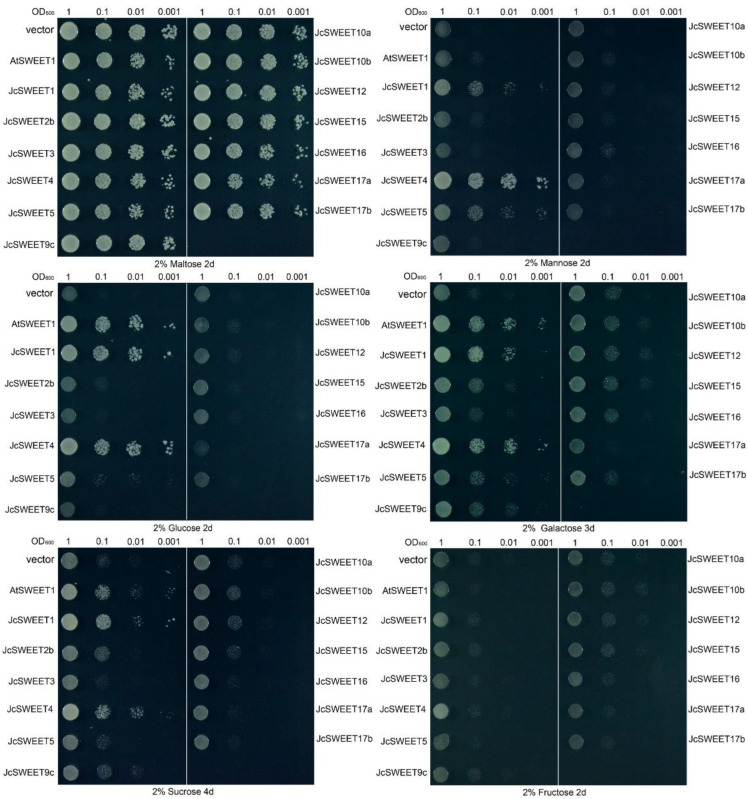
Complementation growth assay in the yeast EBY.VW4000 mutant. Yeast transformants expressing empty vector (negative control), AtSWEET1 and 13 JcSWEETs were grown on media containing 2% maltose, 2% mannose, 2% glucose, 2% galactose, 2% sucrose or 2% fructose. AtSWEET1 was used as positive controls for glucose, galactose and sucrose transport activities.

To obtain cDNAs containing full-length coding sequences of *JcSWEET* genes, total RNA from three-week-old seedlings of physic nut cultivar GZQX0401 was used to perform reverse transcription. As a result, cDNA clones containing full-length coding sequences were obtained for 13 genes. For five genes (*JcSWEET2a*, *JcSWEET6*, *JcSWEET9a*, *JcSWEET9b* and *JcSWEET11*), cDNA clones were not obtained, most likely due to the low level of expression of each gene. Many plant SWEETs have been shown to transport hexoses or sucrose [9]. To examine which substrates could be transported by the JcSWEET proteins, 13 *JcSWEET* genes were expressed in the yeast mutant EBY.VW4000, which lacks endogenous hexose transporters. Accordingly, this mutant yeast can grow on media containing maltose but shows no or slow growth on media containing glucose, fructose, mannose, sucrose or galactose [42]. All transformants with empty vectors and constructs could grow well on synthetic-deficient (SD) media containing 2% maltose, indicating the presence of the expression vector or target gene (Figure 6). Yeast cells expressing either *JcSWEET1* or *JcSWEET4* could grow on media supplemented with mannose, glucose, galactose and sucrose, suggesting that both JcSWEET1 and JcSWEET4 can transport these four sugars in yeast. The expression of *JcSWEET5* effectively restored the growth of EBY.VW4000 on media supplemented with mannose or glucose. However, transformants carrying the remaining genes showed no growth on any of the media, indicating that these ten JcSWEET proteins could not transport these five sugars in yeast using the assay method described here.

### 2.6. Overexpression of JcSWEET16 Causes Early Flowering and Increases Salt Stress Tolerance in A. thaliana

**Figure 7:**
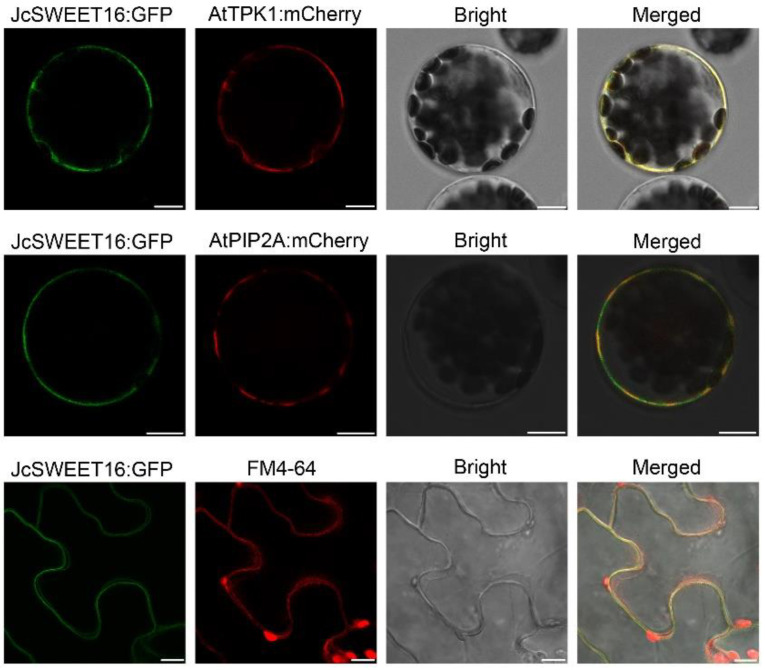
Subcellular localization of JcSWEET16 in *A. thaliana* mesophyll protoplasts and *N. benthamiana* leaves. Top two rows: confocal images of *A. thaliana* mesophyll protoplasts transiently expressing JcSWEET16:GFP fusions and the vacuolar membrane marker AtTPK1:mCherry or the plasma membrane marker AtPIP2A:mCherry, indicating localization to the vacuolar membrane and the plasma membrane. Bottom row: confocal images of *N. benthamiana* epidermal leaf cells transiently expressing JcSWEET16:GFP fusions. Green fluorescence of JcSWEET16:GFP, red fluorescence from FM4-64 labeling the plasma membrane and merged images are shown, indicating localization to the plasma membrane. Scale bar = 10 µm.

To investigate the functions of physic nut *SWEET* genes, several of the genes that demonstrated changes in expression as a result of drought and salinity stress were over-expressed in *A. thaliana* under the control of a CaMV (Capsicum Mottle Virus) 35S promoter. First, we analyzed the subcellular localization of the JcSWEET16 protein. To accomplish this, a JcSWEET16:GFP construct was generated and transiently co-transformed into *A. thaliana* mesophyll protoplasts with a vacuolar membrane marker (AtTPK1:mCherry) or a plasma membrane marker (AtPIP2A:mCherry). Confocal images showed that GFP fluorescence signals of JcSWEET16:GFP overlapped with red fluorescence derived from AtTPK1:mCherry and AtPIP2A:mCherry. To further examine localization, JcSWEET16:GFP was introduced into the epidermal cells of *N. benthamiana* leaves, and the GFP fluorescence was clearly observed on the plasma membrane (Figure 7). These results show that JcSWEET16 was localized not only on the vacuolar membrane but also on the plasma membrane, a pattern similar to the subcellular localization of SlSWEET15 in tomato (*Solanum lycopersicum*) [43].

**Figure 8:**
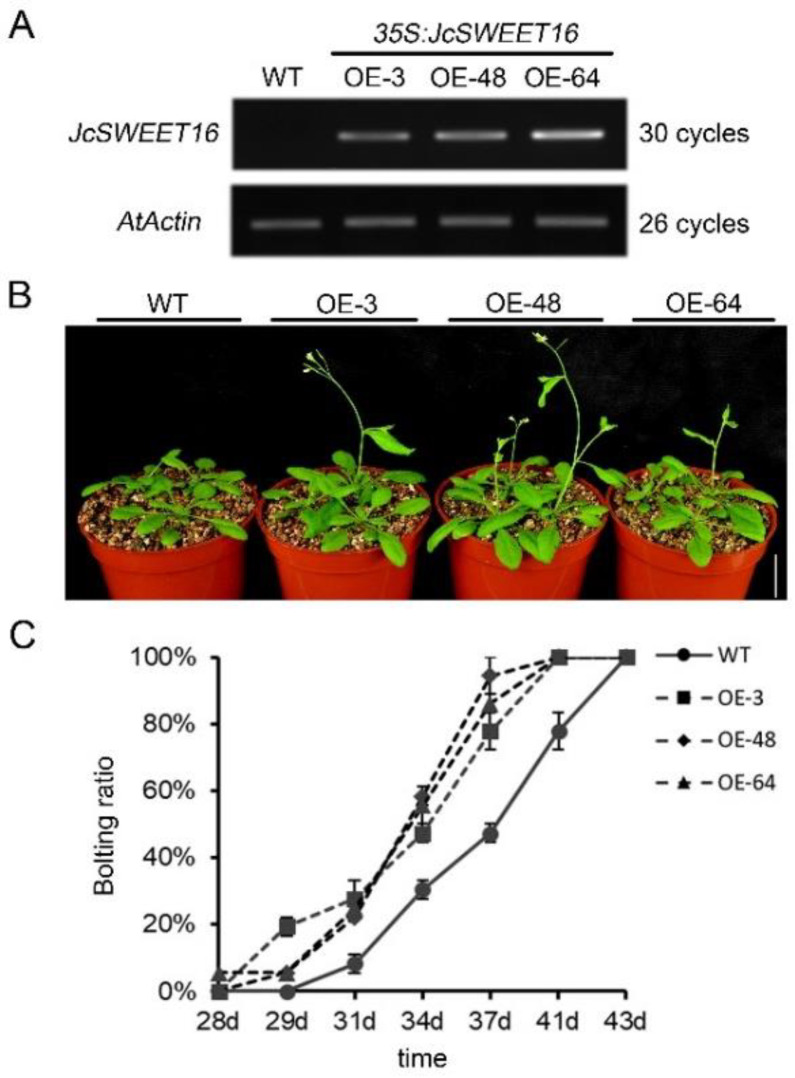
Overexpression of *JcSWEET16* (OE) promotes flowering in *A. thaliana*. (**A**) Relative expression levels of *JcSWEET16* in different transgenic lines measured using semi-quantitative RT-PCR analysis. (**B**) WT plants and OE lines grown for 34 d. (**C**) Flowering time of WT plants and OE lines. At least 18 plants were used for each experiment. Scale bar = 2 cm.

Three independent T3 homozygous overexpressing *JcSWEET16* transgenic lines (OE-3, OE-48 and OE-64) were obtained, and the expression in these lines was examined by semi-quantitative RT-PCR (Figure 8A). Under normal growth conditions, no significant differences were detected between wild-type and transgenic seedlings in terms of plant size or morphology. However, the transgenic lines displayed an early flowering phenotype. After 41 days of growth, all three OE lines had completed the flowering process, while the WT plants exhibited a flowering efficiency of 77.78% (Figure 8B,C). These results indicate that the overexpression of *JcSWEET16* could accelerate flowering in *A. thaliana*.

**Figure 9:**
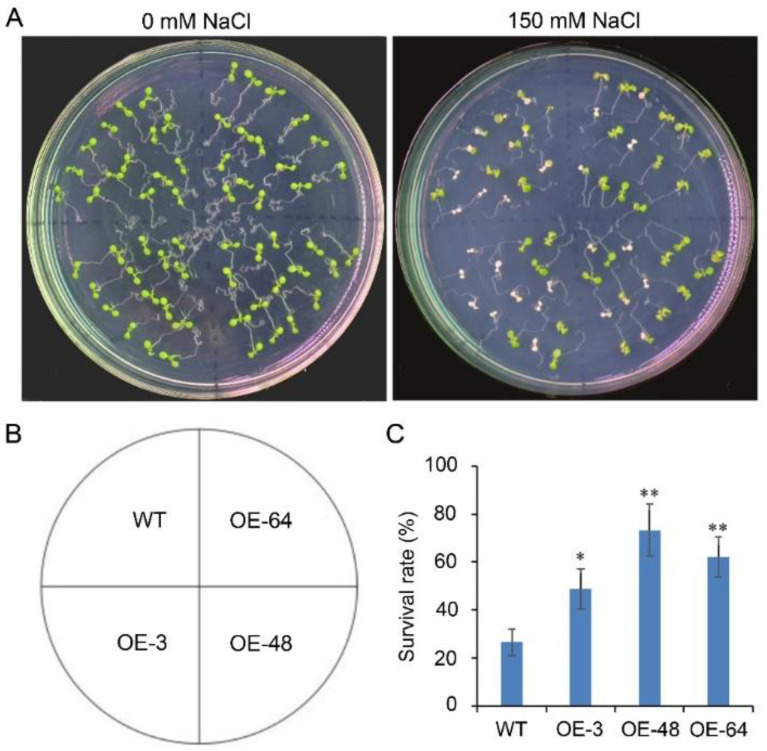
Overexpression of *JcSWEET16* improves saline tolerance in *A. thaliana.* (**A**) Four-day-old seedlings of WT and OE were transferred to 1/2 MS medium supplemented with 150 mM NaCl for 6 days. (**B**) Schematic representation of the seedling position. (**C**) Survival rate of the seedlings after salinity stress. The data shown are means ± SD from three biological experiments. Statistically significant differences were assessed using Student’s *t*-tests (* *p* < 0.05, ** *p* < 0.01).

To gain insight into the function of *JcSWEET16* in response to salinity stress, the same three OE lines were used for salinity treatment. No differences were detected between WT and transgenic lines on a normal 1/2 MS medium, whereas the survival rate of the OE lines was significantly higher than those of WT seedlings when cultivated with 150 mM NaCl (Figure 9). However, there were no significant differences in seedling root length or chlorophyll content between WT and transgenic lines when cultivated with 300 mM Mannitol (Figure S5). These results suggest that the overexpression of *JcSWEET16* in *A. thaliana* could improve saline tolerance.

## 3. Discussion

Over the past two decades, numerous sugar transporters have been discovered in humans, plants, bacteria and fungi and have been demonstrated to play key roles in growth and development, metabolism and homeostasis. The members of the recently identified family of SWEET sugar transporters in eukaryotes contain only seven TMs and can mediate both cellular uptake and efflux [4]. To date, *SWEET* gene family members have been identified in many plant species based on genome-wide analyses. In the present study, a total of 18 putative *SWEET* genes were identified in the physic nut genome; this number is close to those of *A. thaliana* (17) [4], rice (21) [11] and cucumber (17) [44], but fewer than in soybean (52) [24] and wheat (*Triticum aestivuml*) (59) [45]. Polyploidy is an important contributor to plant genome evolution, and many angiosperms have undergone gene duplication within a gene family due to experiencing one or multiple polyploidization events [46,47], which is a genomic event that can explain the large number of *SWEET* genes in the soybean and wheat genomes.

Physic nut *SWEET* genes were classified into four clades according to their phylogenetic relationship (Figure 1), which was in agreement with the classification of *SWEETs* in *A. thaliana* and rice [4,11]. There are eight physic nut *SWEET* genes in clade III, four in clade I, and three in each of clades II and IV (Figure 1). The physic nut has more genes than *A. thaliana* in clades I, III and IV. Gene duplication plays a crucial role in the evolution of higher plants, as it not only expands genome content but also diversifies gene functions to help the organism to adapt to different environmental conditions [48]. A non-random pattern of introns indicates that they were acquired from a progenitor and stabilized through evolution [49]. Gene structure analysis indicates that all *JcSWEET* genes shared a similar exon-intron arrangement (Figure 2). This is similar to the *SWEET* gene structure in *A. thaliana* [4], indicating that angiosperm *SWEET* genes share a common origin and that there has been gene function divergence as gene expansion occurred later during evolution. In addition, tandem duplication events have been observed in the *SWEET* genes of soybean and rice [24]. Four *JcSWEET* genes (*JcSWEET10a*, *10b*, *11* and *12*) were considered to represent tandem duplication, and these duplications are also present in the genomes of castor bean and *A. thaliana* (Figures S3 and S4). These results suggest that the processes giving rise to the expansion of the *JcSWEET* genes in physic nut included both ancient and recent tandem duplication.

In *A. thaliana*, transport substrates for all SWEET family members have been identified, and different clade members have different transport activities: clade I members allow 2-deoxyglucose and sucrose transport, clade II members allow the transport of glucose, clade III is reported to be a sucrose-specific clade and clade IV confers the ability to transport glucose and fructose [9]. In our study, we determined that both JcSWEET1 and JcSWEET4 proteins, from clades I and II, respectively, could mediate the uptake of mannose, sucrose, glucose and galactose in the yeast EBY.VW4000 mutant, while JcSWEET5, which also belongs to clade II, mediated the uptake of mannose and glucose in the assessed yeast mutant background (Figure 6). These results suggest that there may exist some differences in the functions of SWEET proteins among species. Furthermore, SWEET proteins have a broader substrate range in physic nut, indicating that these proteins may have multiple physiological functions and be involved in more complex biological processes. However, in the present study, JcSWEET2b/3/9c/10a/10b/12/15/16/17a/17b showed no sugar transport activity in yeast using the present assay method (Figure 6); this may be because the transport activity of these proteins is not located at the plasma membrane, and further studies are needed.

Plant leaves are the main source organ and play an important role in the synthesis of carbohydrates. Gene expression analysis revealed that *JcSWEET17a* had a relatively high expression level in physic nut leaves (Figure 3). *AtSWEET17* of clade IV is a vacuolar transporter that controls fructose content in *A. thaliana* leaves and roots [50]. In addition, *JcSWEET17a* belongs to clade IV (Figure 1), indicating that it may play similar roles in balancing intracellular hexose homeostasis. Developing seeds are the strongest sink tissues in many plants, and they need a substantial source of carbon for development, which implies that *SWEET* genes may direct a key role in seed development [24]. In *A. thaliana*, *AtSWEET15* is expressed in the seed coat and endosperm and functions in the transfer of sugars from the seed coat to the embryo [16]. The *AtSWEET15* paralog *JcSWEET15* was also found to be significantly expressed in filling-stage seeds (Figure 4), suggesting that this gene may play a similar role to that of *AtSWEET15.* In addition, the duplicates from clade III (*JcSWEET10a* and *10b*) show divergent expression patterns (Figure 3 and Figure S3), suggesting the occurrence of subfunctionalization during the evolutionary process. *AtSWEET9* is a nectary-specific sugar transporter and is essential for nectar production [18]. Both *JcSWEET9a* and *JcSWEET9c* are homologs of *AtSWEET9*, but they have significantly distinct expression patterns. The discrepancy between *AtSWEET9* and *JcSWEET9* is probably due to evolutionary differences at the genome level between these two species.

Recently, studies have shown that *SWEET* genes from clade IV are involved in abiotic stress tolerance and the overexpression of these genes in plants can enhance their tolerance of abiotic stress [26,27,29,51,52,53]. In our study, most of the *JcSWEET* genes showed changes in transcription levels under abiotic stress treatment, including drought and salinity stress (Figure 5A). Both *JcSWEET16* and *JcSWEET17b* are members of clade IV, but they showed opposite expression patterns under drought and salinity treatments. Further studies are needed to confirm whether and how these genes function in response to abiotic stresses. The qRT-PCR analysis showed that the expression of *JcSWEET16* was down-regulated by salinity treatment (Figure 5B). Analysis of the survival rate of the seedlings showed that *JcSWEET16* overexpression could improve salinity tolerance in *A. thaliana*, which was in line with the modified expression levels (Figure 9C). In addition, JcSWEET16 was localized not only at the vacuolar membrane but also at the plasma membrane (Figure 7), in contrast to tonoplast-localized AtSWEET16 [27], indicating that it may function at different stages of growth and development in physic nut. Moreover, the overexpression of *JcSWEET16* in *A. thaliana* could accelerate flowering (Figure 8B,C). Soluble sugar is not only a source of carbon and energy; it also acts as an osmotic regulator [54,55], and transgenic plants that accumulate sucrose in the leaves show more rapid flowering in species such as tomato, potato (*Solanum tuberosum*) and *A. thaliana* [56,57,58]. Whether the overexpression of *JcSWEET16* promotes flowering in *A. thaliana* by affecting sugar metabolism remains to be studied.

## 4. Materials and Methods

### 4.1. Preparation of Plant Materials

Physic nut (*Jatropha curcas* L.) cultivar GZQX0401 was used in this study. After disinfecting with 1:5000 KMnO_4_ solution for 30 min, the seeds were germinated in sand and grown in trays containing a 3:1 mixture of sand and soil in a greenhouse illuminated with natural sunlight in Guangzhou (113.3 ° E, 23.1 ° N). The trays were irrigated with 1.0 L (L) of Hoagland nutrient solution (pH 6.0) once every two days at dusk after the emergence of the first true leaf. Roots, stem cortex, and leaves were sampled at the six-leaf stage (eight weeks after germination). Male and female flowers were sampled in the summer (June 2019), and fresh seeds were sampled in the autumn (September 2020). Sampling at the different stages of seed development was in accordance with previous methods [59].

Exposure to 100 mM NaCl can induce a moderate stress response but is not acutely lethal in physic nut [60]. Indeed, in our previous study, we observed visible signs of leaf chlorosis and defoliation after the treatment [61]. Therefore, stress treatment was begun at the six-leaf stage (eight weeks after germination). For salinity treatment, the seedlings were irrigated with Hoagland solution plus 100 mM NaCl every day. For drought treatment, irrigation was withheld. Leaf samples were collected 7 days after the onset of drought stress and salinity stress. The details of salinity and drought treatment were in accordance with previous methods [40,41]. All samples were frozen immediately in liquid nitrogen and stored at −80 °C before qRT-PCR analysis. Three independent biological replicates were performed for each analysis.

### 4.2. Sequence Database Searches and Gene Cloning

To identify physic nut *SWEET* genes, we searched for *SWEET* genes in the physic nut genome database of the Kazusa DNA Research Institute [37] and our genome database [36] using *A. thaliana* and rice SWEET protein sequences as queries. SWEET protein sequences from *A. thaliana* and rice were obtained from the *A. thaliana* genome database TAIR (https://www.arabidopsis.org/ (accessed on 13 February 2020)) and the rice genome annotation database (http://rice.uga.edu/ (accessed on 23 February 2020)), respectively. Sequences giving E values of less than 1 × 10^−10^ were selected for further analysis, and all *JcSWEET* sequences were revised based on information in the expressed sequence tag (EST) database (http://www.ncbi.nlm.nih.gov/ (accessed on 9 January 2022)) and our physic nut and *Jatropha integerrima* EST datasets (SRA197144 and SRA197148 in GenBank). The Pfam program (http://pfam.xfam.org/ (accessed on 9 January 2022)) was used to confirm the presence of the MtN3 domain of all putative SWEET proteins. All target sequences were subsequently used to clone the full-length *JcSWEET* genes. The cDNA from three-week-old seedlings of physic nut was used as a template for amplifying the *JcSWEET* genes with the specific primers listed in Table S2. The PCR products were cloned into the pMD18-T vector (TaKaRa) and then sequenced.

### 4.3. Sequence Analysis and Phylogenetic Tree Construction

Transmembrane domains in JcSWEET proteins were predicted using TMHMM Server v. 2.0 [62]. Information, including accession number and MtN3/saliva (PQ-loop repeat) domain position in the *JcSWEET* genes, was acquired from NCBI. Multiple sequence alignments of protein sequences were performed by DNAMAN. The exon/intron structures of *JcSWEET* genes were analyzed using Gene Structure Display Server (GSDS, http://gsds.gao-lab.org/ (accessed on 19 January 2022)) [63] by comparing the coding sequences and the corresponding genomic sequences. Chromosome localization was performed using MapChart 2.32, based on the linkage map constructed in our previous study [36,64].

To analyze the relationships of the *SWEET* genes in physic nut, the full-length JcSWEET protein sequences and SWEET protein sequences from *A. thaliana* and rice were used to generate a phylogenetic tree. The tree was constructed using MEGA 5.0 by the neighbor-joining (NJ) method with default settings, and the results were displayed with iTOL (http://itol.embl.de/ (accessed on 8 January 2022)). Amino acid sequences of the SWEET proteins used for the analysis are listed in Table S3.

### 4.4. RNA Isolation and qRT-PCR

Total RNA was extracted from the samples, and the first strand cDNA was synthesized as previously described [65]. All qRT-PCR experiments were run on a LightCycler^®^ 480 Real-Time PCR System (Roche, Basel, Switzerland). The reference gene *JcActin* was used as the internal control, and the expression levels were calculated using the 2^−∆CT^ method. All specific primer sequences are listed in Table S2.

### 4.5. Plasmid Construction and Complementation Assays in Yeast

pMD18-T-JcSWEET clones were used as templates for amplifying the coding regions of the *JcSWEET* genes with *XhoI* and *Bam*HI cleavage sites, and the amplified fragments were cloned into the yeast expression vector pDR195. The specific primers are listed in Table S2. Then, all resulting constructs were transformed into the hexose transport-deficient yeast strain EBY.VW4000. For the yeast complementation growth assay, serial dilutions (1, 0.1, 0.01 and 0.001) of all desired transformants were spotted on synthetic deficient media containing 2% maltose (as the control), glucose, galactose, mannose, fructose or sucrose. The plates were photographed after 2–4 days of growth at 30 °C.

### 4.6. Subcellular Localization of JcSWEET16

The coding sequence of the *JcSWEET* gene (without stop codons) was amplified with the specific primer pair 5′-GGTACCATGGCTAGCTTAAGCTTC-3′ and 5′- GTCGACAAGATCATTATCAACTTT-3′ and then cloned into the 35S:GFP vector. *A. thaliana* mesophyll protoplasts were isolated and transformed with the resulting construct as previously described [66]. For transient expression in *N. benthamiana* leaves, the resulting binary vector was transformed into *A. tumefaciens* strain GV3101 and used to infect *N. benthamiana* epidermal cells. To determine the positions of inner membranes, AtPIP2A:mCherry and AtTPK1:mCherry were used as markers for the plasma membrane and vacuolar membrane, respectively. For FM4-64 staining, tobacco leaves were incubated in 4 μM FM4-64 for 15 min before observation. Fluorescence was observed on a Leica TCS SP8 confocal laser scanning microscope.

### 4.7. Plant Transformation and Salinity Treatment of Transgenic A. thaliana

In order to get transgenic *A. thaliana*, the expression vector 35S:JcSWEET16:GFP was constructed and was transformed into *A. tumefaciens* strain GV3101. Subsequently, the resulting transformants were used to infect *A. thaliana* plants (Col-0 ecotype) as described previously [67]. Seeds from single insertion homozygous transgenic lines were chosen for the subsequent analysis. The expression levels in the transgenic lines were determined by semi-quantitative RT-PCR with the specific primers 5′-GCACCGTCTTCCAATTCGTT-3′ and 5′-ACGCCTCCATTGAGAAACAG-3′, and the reference gene *AtActin* (AT3G18780) was used as the internal control (AtActin-F: AGATGCCCAGAAGTCTTGTTCC, AtActin-R: TTTGCTCATACGGTCAGCGATA).

After surface disinfection, the seeds of transgenic and wild-type lines were incubated for 2 days in the dark at 4 °C. Plants were grown under a long-day photoperiod (16 h light/8 h dark) at 22 ± 2 °C in a growth chamber. Flowering time was scored by observing the bolting ratio, and a bolting height of 0.5 cm was taken to indicate bolting. For salinity treatment, transgenic and wild-type seeds were surface-sterilized and sown on one half-strength Murashige and Skoog (MS) medium (pH 5.7, KOH) containing 1.0% (*w*/*v)* sucrose and 1.0% agar (*w*/*v*). The plates were incubated in the dark at 4 °C for 2 days and then grown in a growth chamber with a long-day photoperiod (16 h light/8 h dark) at 22 ± 2 °C. After growth for 4 days, similarly sized seedlings were transferred to new 1/2 MS medium supplemented with 150 mM NaCl as described previously [68]. The survival rate of the seedling was counted, and photos were taken after 6 days of treatments.

## Data Availability

Not applicable.

## Supplementary Materials

The following supporting information can be downloaded at: https://www.mdpi.com/article/10.3390/ijms23105391/s1.
