# Supplementary material for: Genome-Wide Identification, Expression Patterns and Sugar Transport of the Physic Nut SWEET Gene Family and a Functional Analysis of JcSWEET16 in Arabidopsis
Source: Int J Mol Sci. 2022 May 12;23(10):5391. doi: 10.3390/ijms23105391 (PMC9142063; doi:10.3390/ijms23105391)
Supplement: Supplementary file 1 [file ijms-23-05391-s001.zip › Figure S1.pdf]

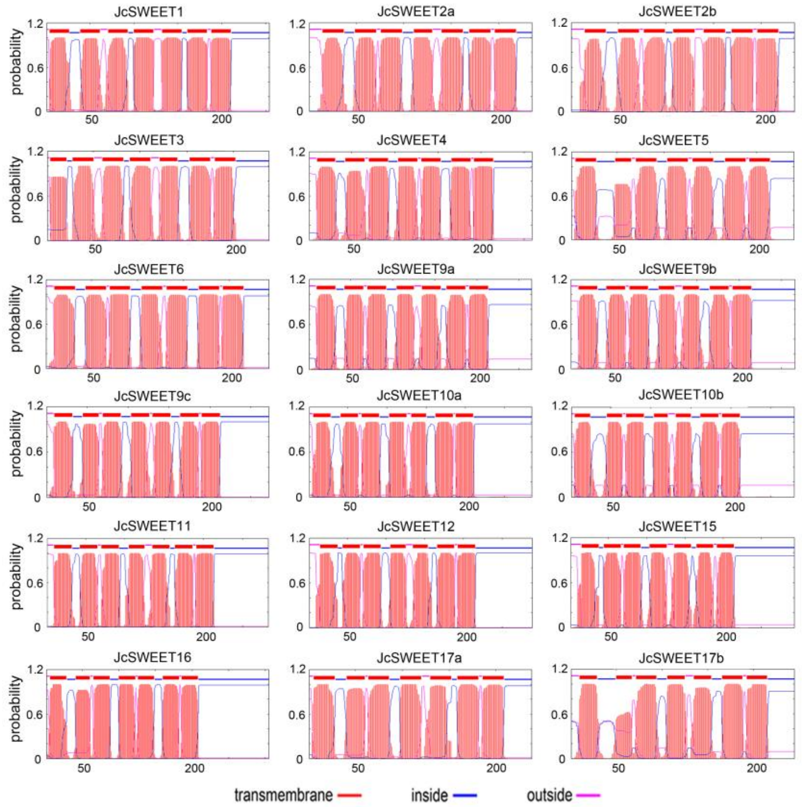

Figure S1. Transmembrane domains of JcSWEET proteins. Transmembrane domains in JcSWEET proteins were predicted using TMHMM Server v. 2.0. The position of N- and C-terminal domains of the protein were indicated by blue or pink lines.
