# Supplementary material for: Genome-Wide Identification, Expression Patterns and Sugar Transport of the Physic Nut SWEET Gene Family and a Functional Analysis of JcSWEET16 in Arabidopsis
Source: Int J Mol Sci. 2022 May 12;23(10):5391. doi: 10.3390/ijms23105391 (PMC9142063; doi:10.3390/ijms23105391)
Supplement: Supplementary file 1 [file ijms-23-05391-s001.zip › Figure S2.pdf]

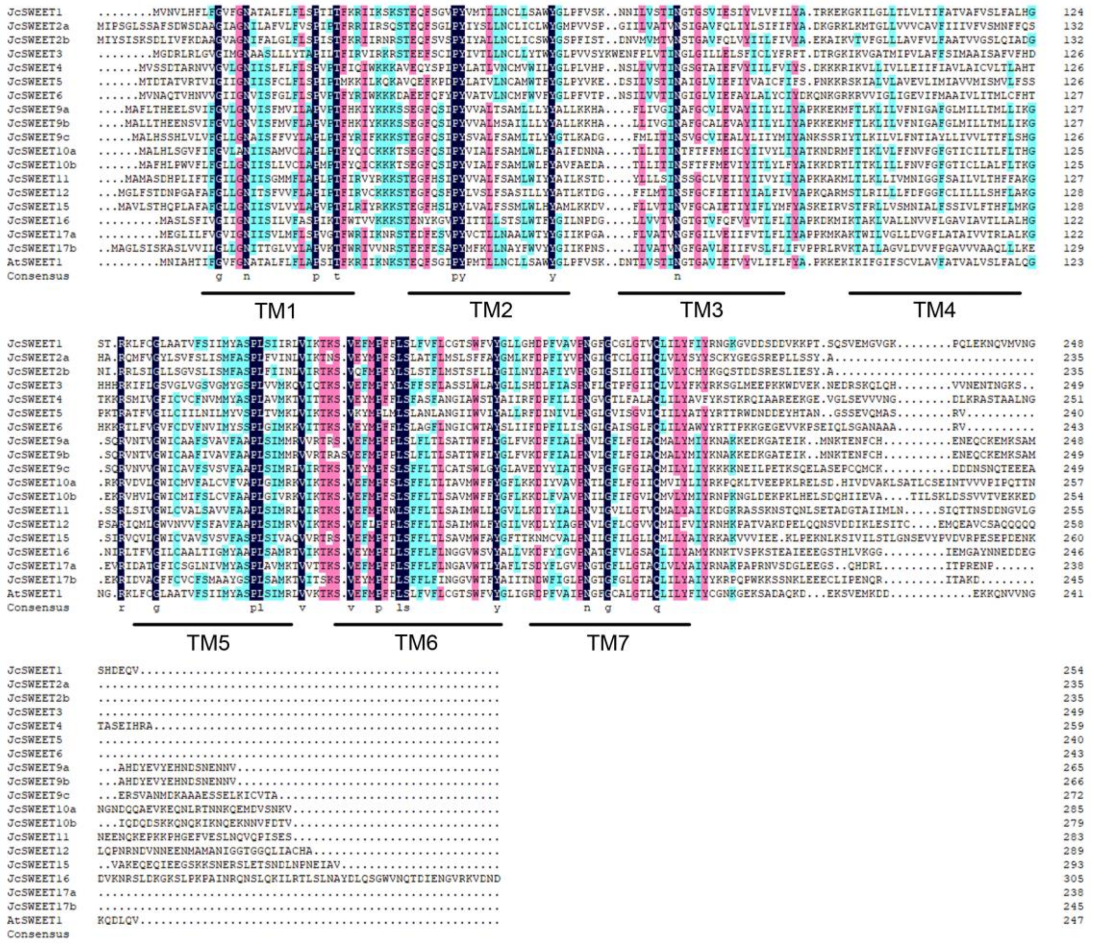

Figure S2. Multiple sequence alignment of JcSWEET proteins. Alignment of protein sequences of 18 JcSWEETs and AtSWEET1 transporters. Highly conserved residues are indicated in colour. Seven transmembrane domains are indicated with TM.
