# Supplementary material for: Genome-Wide Identification, Expression Patterns and Sugar Transport of the Physic Nut SWEET Gene Family and a Functional Analysis of JcSWEET16 in Arabidopsis
Source: Int J Mol Sci. 2022 May 12;23(10):5391. doi: 10.3390/ijms23105391 (PMC9142063; doi:10.3390/ijms23105391)
Supplement: Supplementary file 1 [file ijms-23-05391-s001.zip › Figure S3.pdf]

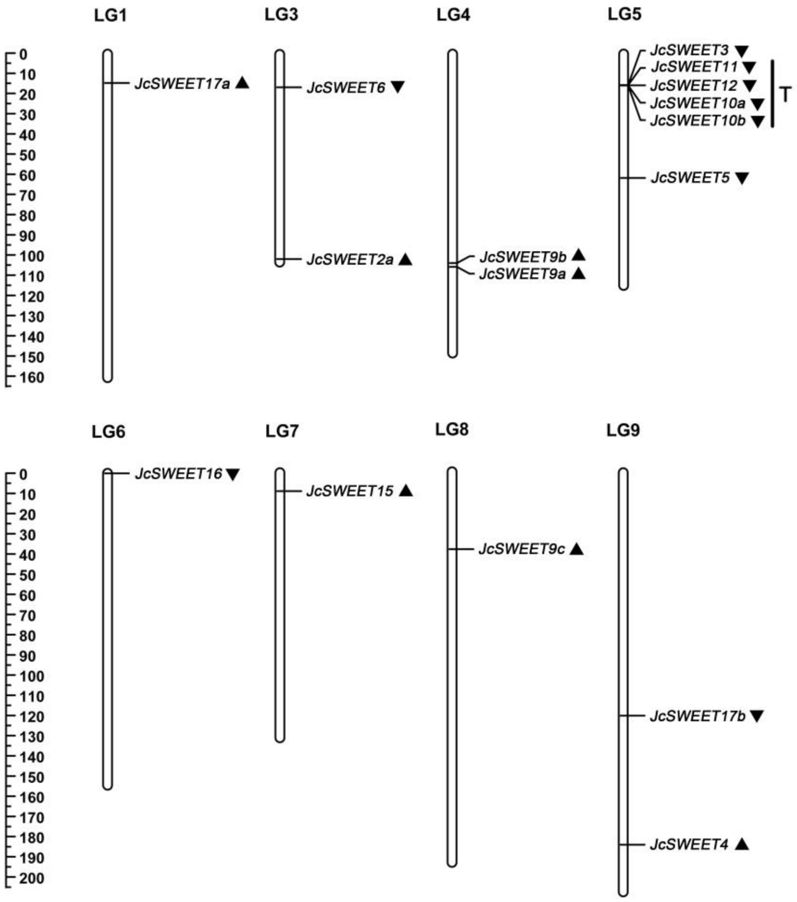

Figure S3. Distribution of *JcSWEET* genes on linkage groups (LGs). Of the 18 predicted *JcSWEET* genes, 16 were mapped to the 11 LGs. The scale is in centimorgans. T, tandem duplication. The arrowheads show the direction of transcription.
