# Supplementary material for: Genome-Wide Identification, Expression Patterns and Sugar Transport of the Physic Nut SWEET Gene Family and a Functional Analysis of JcSWEET16 in Arabidopsis
Source: Int J Mol Sci. 2022 May 12;23(10):5391. doi: 10.3390/ijms23105391 (PMC9142063; doi:10.3390/ijms23105391)
Supplement: Supplementary file 1 [file ijms-23-05391-s001.zip › Figure S4.pdf]

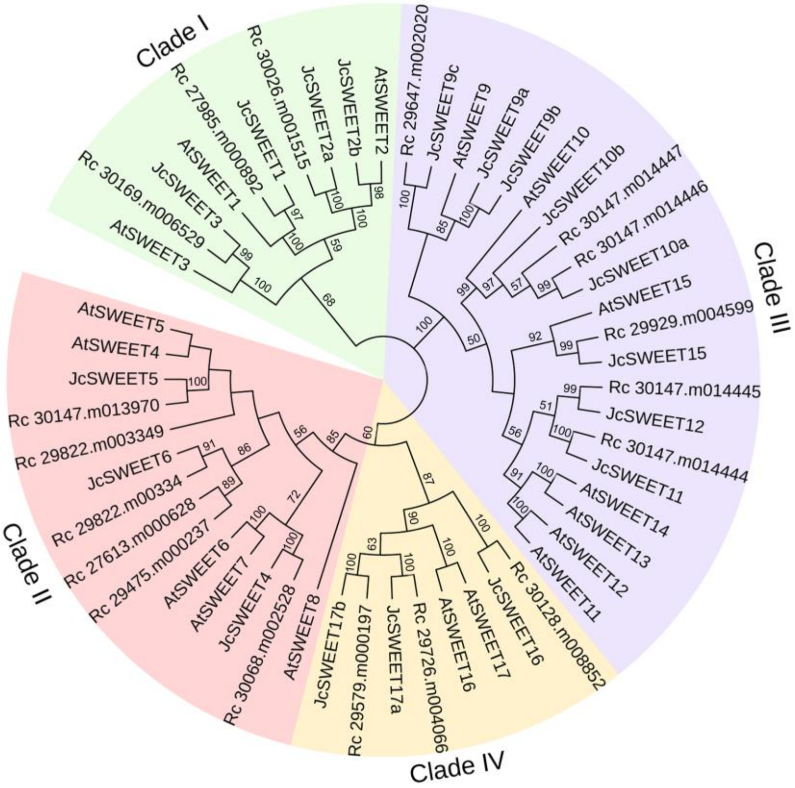

Figure S4. Phylogenetic relationships of the *SWEET* family genes in physic nut, castor bean, and *A. thaliana*. The sequences of the SWEET proteins from the above three plant species were aligned by CLUSTAL_X, and the phylogenetic tree was constructed by using MEGA 5.0 and neighbor-joining (NJ) method with default setting. Jc, *Jatropha curcas* ; At, *A. thaliana*; Rc, *Ricinus communis.*
