# Supplementary material for: Genome-Wide Identification, Expression Patterns and Sugar Transport of the Physic Nut SWEET Gene Family and a Functional Analysis of JcSWEET16 in Arabidopsis
Source: Int J Mol Sci. 2022 May 12;23(10):5391. doi: 10.3390/ijms23105391 (PMC9142063; doi:10.3390/ijms23105391)
Supplement: Supplementary file 1 [file ijms-23-05391-s001.zip › Figure S5.pdf]

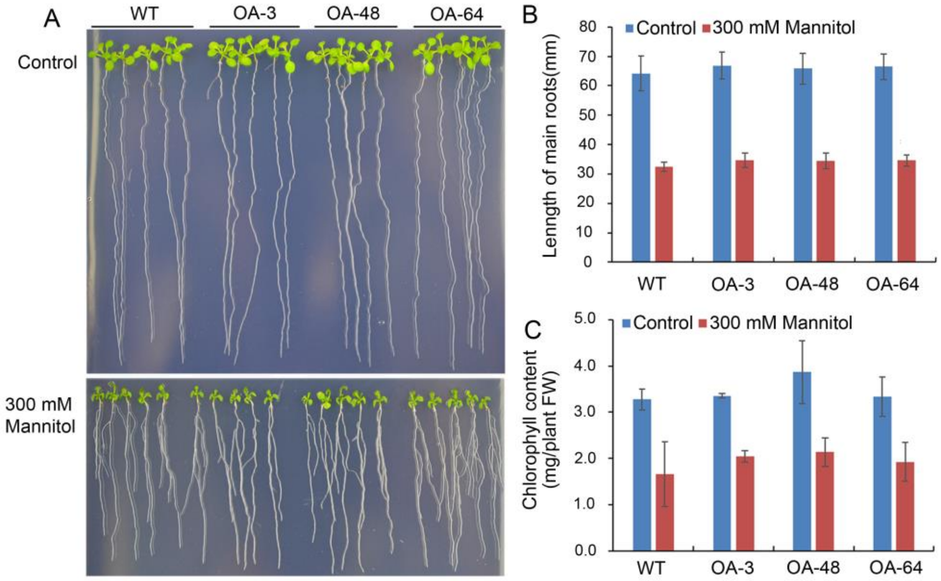

Figure S5. Effects of drought stress on overexpression of *JcSWEET16* in *A. thaliana*. (A) Four-day-old seedlings of WT and OE were transferred to 1/2 MS medium supplemented with 300 mM Mannitol for 10 days. Root lengths (B) and chlorophyll contents (C) of the seedlings. The data shown are means ± SD from three biological experiments.
