# Supplementary material for: Genome-Wide Identification, Expression Patterns and Sugar Transport of the Physic Nut SWEET Gene Family and a Functional Analysis of JcSWEET16 in Arabidopsis
Source: Int J Mol Sci. 2022 May 12;23(10):5391. doi: 10.3390/ijms23105391 (PMC9142063; doi:10.3390/ijms23105391)
Supplement: Supplementary file 1 [file ijms-23-05391-s001.zip › Table S1.pdf]

Table S1 Information about physic nut *SWEET* genes.

Gene name	Accession	ORF length (bp)	AA	TMs	MtN3 (PQ-loop repeat) domain position		*Arabidopsis* homologous gene
JcSWEET1	XP_012066096.1	765	254	7	7-96	129-215	AT1G21460
JcSWEET2a	XP_012091868.1	708	235	7	15-104	137-222	AT3G14770
JcSWEET2b	XP_012075503.1	708	235	7	15-104	137-223	AT3G14770
JcSWEET3	XP_012090112.1	750	249	7	8-98	132-217	AT5G53190
JcSWEET4	XP_012086673.1	780	259	7	9-98	132-218	AT3G28007
JcSWEET5	XP_020537596.1	723	240	7	9-98	132-218	AT5G62850
JcSWEET6	XP_012081801.1	732	243	7	9-98	133-219	AT4G10850
JcSWEET9a	XP_012078372.1	798	265	7	11-98	132-218	AT2G39060
JcSWEET9b	XP_037497077.1	801	266	7	11-98	131-219	AT2G39060
JcSWEET9c	XP_012083783.1	819	272	7	10-97	131-217	AT2G39060
JcSWEET10a	XP_012090633.1	858	285	7	9-96	130-216	AT5G50790
JcSWEET10b	XP_012090634.2	840	279	7	9-96	130-216	AT5G50790
JcSWEET11	XP_012090787.2	852	283	7	11-98	132-218	AT3G48740
JcSWEET12	XP_012090788.1	870	289	7	12-99	134-220	AT5G23660
JcSWEET15	XP_012085745.1	882	293	7	12-99	133-219	AT5G13170
JcSWEET16	XP_012088980.1	918	305	7	6-93	127-213	AT3G16690
JcSWEET17a	XP_012083966.1	717	238	7	6-93	127-213	AT4G15920
JcSWEET17b	XP_012086437.1	738	245	7	13-100	134-220	AT4G15920

Data in this table are from NCBI database. ORF, open reading frame ; AA, amino acid; TMs, transmembrane domains, MtN3, MtN3 saliva family (PFAM database code PF03083; http://pfam.xfam.org)
