# Supplementary material for: Genome-Wide Identification, Expression Patterns and Sugar Transport of the Physic Nut SWEET Gene Family and a Functional Analysis of JcSWEET16 in Arabidopsis
Source: Int J Mol Sci. 2022 May 12;23(10):5391. doi: 10.3390/ijms23105391 (PMC9142063; doi:10.3390/ijms23105391)
Supplement: Supplementary file 1 [file ijms-23-05391-s001.zip › Table S3.pdf]

Supplementary Table S3 Amino acid sequences used to generate the phylogenetic tree.

> JcSWEET1
MVNVLHFLFGVFGNATALFLFLSPTITFKRIIKSKSTEQFSGVPYVMTLLNCLLSAWYGLPFV
SKNNILVSTINGTGSVIESIYVLVFILYATRKEKGKILGLLTLVLTIFATVAFVSLFALHGSTRKLF
CGLAATVFSIIMYASPLSIIRLVIKTKSVEFMPFFLSLFVFLCGTSWFVYGLLGHDPFVAVPNGF
GCGLGTVQLILYFIYRNGKGVDDSDDVKKPTSQSVEMGVGKPQLEKNQVMVNGSHDEQV

> JcSWEET2a
MIPSGLSSAFSDWSDAAGIAGNILAFVLFVSPIPTFRRIIRSQSTEQFSGLPYIYSLLNCLICLWY
GMPVVSPGIILVATVNSIGAVFQLIYLSIFIFYADKGRKLKMTGLLVVVCAVFIIIVFVSMNFFQ
SHARQMFVGYLSVFSLISMFASPLFVINLVIKTNSVEYMPFSLSLATFLMSLSFFAYGMLKFDP
FIYVPNGIGTCLGILQLVLYSYYSCKYGEGSREPLLSSYA

> JcSWEET2b
MIYSISKSDLIVFKDAAGVAGNIFALGLFLSPISTFKRIIRNRSTEQFSVSPYIYALLNCLICSWY
GSPFISTDNVMVMTVNSTGAVFQLVYIILFIVYAEKAIKVTVFGLLLAVFVLFAATVVGSLQIA
DGNIRRLSIGLLSGVSLISMFASPLFIINLVIRTKSVQFMPFYLSLSTFLMSTSFLLYGILNYDAFI
YVPNGIGSILGITQLVLYCHYKGQSTDDSRESLIESYA

> JcSWEET3
MGDRLRLGVGIMGNAASLLLYTAPILTFIRVIRKRSTEEFSCIPYIVTLLNCLLYTWYGLPVVS
YKWENFPLVTINGLGILLELSFICLYFRFTDTRGKIKVGATMIPVLAFFSIMAAISAFVFHDHH
HRKIFLGSVGLVGSVGMYGSPLVVMKQVIQTKSVEFMPFYLSFFSFLASSLWLAYGLLSHDLF
IASPNFLGTPFGIIQLVLYFKYRKSGLMEEPKKWDVEKNEDRSKQLQHVVNENTNGKS

> JcSWEET4
MVSSDTARNVVGVLGNIISLFLFLSPVPTFIQIWKKKAVEQYSPIPYLATLVNCMVWILYGLPL
VHPNSLLVVTINGSGTAIEFVYIILFVIYSDKKKRIKVLLIVLLEIIFIAVLAICVLTLAHTTKKRS
MIVGFICVCFNVMMYASPLAVMKTVITTKSVEYMPFFLSFASFANGIAWSTYAIIRFDPFILIP
NGVGTLFALAQLILYAVFYKSTKRQIAAREEKGEVGLSEVVVNGDLKRASTAALNGTASEIH
RA

> JcSWEET5
MTDTATVRTVIGIIGNIISFCLFLSPIPTMKKILKQKAVQEFKPDPYLATVLNCAMWTFYGLP
YVKEDSILVSTINAIGLVIEFIYVAICFIFSPNKKRSKIALVLAVEVLIMIAVVMISMVLFSSPKTR
ATFVGILCIILNILMYVSPLTVMKLVIKTKSVKYMPLMLSLANLANGIIWVIYALLRFDINIVL
PNGLGVISGVIQIILYATYYRTTRWDNDDEYHTANGSSEVQMASRV

> JcSWEET6
MVNAQTVHNVVGIIGNVISFGLFLSPVPTFYRIWKKKDAEEFQFYPYVATVLNCMFWVFYG
LPFVTPNSILVVTINGIGLVIEFAYLALYCIYDKQNKGRKRVVIGLIGEVIFMAAIVLITMLCFH
THKKRTLFVGVFCDVFNVIMYSSPLGIMKKVITTKSVEYMPFFLSLAGFLNGICWTAYSLIIFD

PFILISNGLGAISGLFQLILYAWYYRTTPKKGEGEVVKPSEIQLSGANAAARV

> JcSWEET9a
MAFLTHEELSVIFGVLGNVISFMVILAPVPTFHKIYKKKSSEGFQSIPYVVALTSAMLLLYYAL
LKKHAFLIVGINAFGCVLEVAYIILYLIYAPKKEKMFTLKLILVFNIGAFGLMILLTMLLIKGSQ
RVNTVGWICAAFSVAVFAAPLSIMMRVVRTRSVEFMPFPLSLFLTLSATTWFLYGLFVKDFFI
ALPNVLGFLFGIAQMALYMIYKNAKKEDKGATEIKMNKTENFCHENEQCKEMKSAMAH
DYEVYEHNDSNENNV

> JcSWEET9b
MALLTHEENSVIFGVLGNVISFMVFLAPVPTFHKIYKKKSSEGFQSIPYVVALMSAILLLYYAL
LKKHALLIVGINAFGCALEVAYIILYLIYAPKKEKMFTLKLILVFNIGAFGLMILLTMLLIKGSQ
RVNTVGWICAAFIVAVFAAPLSIMMRVVRTRASVEFMPFPLSLFLTLSATTWFLYGLFVKDFF
IALPNVLGFLFGIAQMALYMIYKNAKKEDKGATEIKMNKTENFCHENEQCKEMKSAMAH
DYEVYEHNDSNENNV

> JcSWEET9c
MALHSSHLVLVFGLLGNAISFFVYLAPLPTFYRIFKKKSTEGFQSIPYSVALFSAMLTLYYGTL
KADGFMLITINSVGCVIEALYLIIYMIYANKSSRIYTLKILVLFNTIAYLLIVVLTTFLSHGSQRV
NVVGWICAVFSVSVFAAPLSIMRLVIRTKSVEYMPFSLSFFLTLCATSWLGYGLAVEDYYIATP
NVFGFGFGIAQMILYLIYKKKKNEILPETKSQELASEPCQMCKDDDNSNQTEEEAERSVAN
MDKAAAESSELKICVTA

> JcSWEET10a
MALHLSGVFIFGVLANIISAMVCLAPLPTFYQICKKKTSEGFQSIPYVIALFSAMLWLFYAIFD
NNATLLITINTFTFFMEICYIIVYLIYATKNDRMFTIKLVLFFNVFGFGTICILTLFLTHGRKRVD
VLGWICMVFALCVFVAPLGIMRKVIKTKSVEFMPFSLSFFLTLTAVMWFFYGFLKKDIYVAVP
NTLGFLFGIIQMVIYLIYRKPQKLTVEEPKLRELSDHIVDVAKLSATLCSEINTVVVPIPQTTN
NGNDQQAEVKEQNLRTNNKQEMDVSNKV

> JcSWEET10b
MAFHLPWVFLFGLLGNIISLLVCLAPMPTFYQICKKKTSEGFQSIPYVIALFSAMLWLFYAVF
AEDATLLITINSFTFFMEVIYITLYLFYAIKKDRTLTTKLILLFNVFGFGTICLLALFLTKGEKRV
HVLGWICMIFSLCVFAAPLGIVRKVIKTKSVEFMPFSLSFFLTLSAVMWFFYGFLKKDLFVAV
PNILGFIFGVLQMVLYMIYRNPKNGLDEKPKLHELSDQHIIEVATILSKLDSSVVTVEKKEDI
QDQDSKKQNQKIKNQEKNNVFDTV

> JcSWEET11
MAMASDHPLIFTFGILGNIISGMMFLAPLPTFIRVYRKKSTEGFHSIPYVVALFSAMLWIYYAI
LKSTDYLLLSINSSGCLVEIIYIIVYIIYAPKKAKMLTLKLLIVMNIGGFSAILVLTHFFAKGSSRL
SIVGWLCVALSAVVFAAPLSIMRLVIRTKSVEFMPFWLSFFLTLSAIMWLLYGVLLKDIYIALP
NVIGVLLGTVQMALYAIYKDGKRASSKNSTQNLSETADGTAIIMLNSIQTTNSDDNGVLGN
EENQKEPKKPHGEFVESLNQVQPISES

> JcSWEET12
MGLFSTDNPGAFAFGLLGNITSFVVFLAPIPTFIRVCKKKSTEGFQSFPYLVSLFSASILLYYATL
KTDGFFLMTINSFGCFIETIYIALFIVYAPKQARMSTLRILLLFDFGGFCLILLLSHFLAKGPSA
RIQMLGWVNVVFSFAVFAAPLSIMRVVIKTKSVEFLPFPLSFFLTLSAIMWLFYGILVKDLYIA
GPNVLGFLCGVVQMILFVIYRNHKPATVAKDPELQQNSVDDIKLESITCEMQEAVCSAQQQ
QQLQPNRNDVNNEENMAMANIGGTGGQLIACHA

> JcSWEET15
MAVLSTHQPLAFAFGLLGNIISVLVYLAPVPTFLRIYRKKSTEGFHSLPYLVALFSSMLWLHY
AMLKKDVFLLVTINVFGCAIETIYIFLYMFYASKEIRVSTFRLLVSMNIALFSSIVLFTHFLMKG
SIRVQVLGWICVAVSVSVFASPLSIVAQVVRTRSVEFMPFTLSFFLTLSAVMWFAYGFTTKNM
CVALPNILGFILGLLQMLLYAIYRKAKVVVIEEKLPEKNLKSIVILSTLGNSEVYPVDVRPESEP
DENKVAKEQEQIEEGSKKSNERSLETSNDLNPNEIAV

> JcSWEET16
MASLSFIVGIIGNIISILVFASPIKTFWTVVKKKSTENYKGVPYITTLLSTSLWTFYGILNPDGLL
VVTVNGTGTVFQFVYVTLFLIYAPKDKMIKTAKLVALLNVVFLGAVIAVTLLALHGNIRLTF
VGILCAALTIGMYAAPLSAMRTVIKTKSVEYMPFLLSFFLFLNGGVWSVYALLVKDFYIGVP
NATGFVLGSAQLILYAMYKNKTVSPKSTEAIEEEGSTHLVKGGIEMGAYNNEDDEGDVKNR
SLDKGKSLPKPAINRQNSLQKILRTLSLNAYDLQSGWVNQTDIENGVRKVDNDL

> JcSWEET17a
MEGLILFVGVIGNIISVLMFLSPVGTFWRIIKNRSTEDFESVPYVCTLLNAALWTYYGIIKPGA
FLVATVNGFGILVEIIFVTLFLIYAPPKMKAKTWILVGLLDVGFLATAIVVTRLALKGEVRIDA
TGFICSGLNIVMYASPLAVMKTVVTTKSVEFMPFFLSFFLFLNGAVWTLYAFLTSDYFLGVPN
GTGFLLGTAQLVLYAIYRNAKPAPRNVSDGLEEGSQHDRLITPRENP

> JcSWEET17b
MAGLSISKASLVVILGLLGNITTGLVYLAPVKTFWRIVVNRSTEEFESAPYMFKLLNAYFWVY
YGIIKPNSILVATVNGFGAVLEIIFVSLFLIFVPPRLRVKTAILAGVLDVVFPGAVVVAAQLLLK
EEKRIDVAGFFCVCFSMAAYGSPLSAMKTVITSKSVEYMPFLLSFFLFINGGVWTFYAIITND
WFIGLPNGTGFGLGTAQLILYAIYYKRPQPWKKSSNKLEEECLIPENQRITAKD

> AtSWEET1
MNIAHTIFGVFGNATALFLFLAPSITFKRIIKNKSTEQFSGIPYPMTLLNCLLSAWYGLPFVSK
DNTLVSTINGTGAVIETVYVLIFLFYAPKKEKIKIFGIFSCVLAVFATVALVSLFALQGNGRKLF
CGLAATVFSIIMYASPLSIMRLVVKTKSVEFMPFFLSLFVFLCGTSWFVYGLIGRDPFVAIPNG
FGCALGTLQLILYFIYCGNKGEKSADAQKDEKSVEMKDDEKKQNVVNGKQDLQV

> AtSWEET2
MDVFAFNASLSMCKDVAGIAGNIFAFGLFVSPMPTFRRIMRNKSTEQFSGLPYIYALLNCLIC
LWYGTPFISHSNAMLMTVNSVGATFQLCYIILFIMHTDKKNKMKMLGLLFVVFAVVGVIVA
GSLQIPDQLTRWYFVGFLSCGSLVSMFASPLFVINLVIRTKSVEFMPFYLSLSTFLMSASFLLYG
LFNSDAFVYTPNGIGTILGIVQLALYCYYHRNSIEEETKEPLIVSYV

> AtSWEET3
MGDKLRLSIGILGNGASLLLYTAPIVTFSRVFKKKSTEEFSCFPYVMTLFNCLIYTWYGLPIVS
HLWENLPLVTINGVGILLESIFIFIYFYYASPKEKIKVGVTFVPVIVGFGLTTAISALVFDDHRH
RKSFVGSVGLVASISMYGSPLVVMKKVIETRSVEYMPFYLSFFSFLASSLWLAYGLLSHDLFLA
SPNMVATPLGILQLILYFKYKNKKDLAPTTMVITKRNDHDDKNKATLEFVVDVDRNSDTN
EKNSNNASSI

> AtSWEET4
MVNATVARNIAGICGNVISLFLFLSPIPTFITIYKKKKVEEYKADPYLATVLNCALWVFYGLP
MVQPDSLLVITINGTGLAIELVYLAIFFFFSPTSRKVKVGLWLIGEMVFVGIVATCTLLLFHTH
NQRSSFVGIFCVIFVSLMYIAPLTIMSKVIKTKSVKYMPFSLSLANFLNGVVWVIYALIKFDLFI
LIGNGLGTVSGAVQLILYACYYKTTPKDDEDEEDEENLSKVNSQLQLSGNSGQAKRVSA

> AtSWEET5
MTDPHTARTIVGIVGNVISFGLFCAPIPTMVKIWKMKSVSEFKPDPYVATVLNCMMWTFYG
LPFVQPDSLLVITINGTGLFMELVYVTIFFVFATSPVRRKITIAMVIEVIFMAVVIFCTMYFLHT
TKQRSMLIGILCIVFNVIMYAAPLTVMKLVIKTKSVKYMPFFLSLANFMNGVVWVIYACLKF
DPYILIPNGLGSLSGIIQLIIYITYYKTTNWNDDDEDKEKRYSNAGIELGQA

> AtSWEET6
MVHEQLNLIRKIVGILGNFISLCLFLSPTPTFIHIVKKKSVEKYSPLPYLATLLNCLVRALYGLP
MVHPDSTLLVTISGIGITIEIVFLTIFFVFCGRQQHRLVISAVLTVQVVFVATLAVLVLTLEHTTD
QRTISVGIVSCVFNAMMYASPLSVMKMVIKTKSLEFMPFLLSVVGFLNAGVWTIYGFVPFDP
FLAIPNGIGCVFGLVQLILYGTYYKSTKGIMEERKNRLGYVGEVGLSNAIAQTEPENIPYLNK
RVSGV

> AtSWEET7
MVFAHLNLLRKIVGIIGNFIALCLFLSPTPTFVRIVKKKSVEEYSPIPYLATLINCLVWVLYGLP
TVHPDSTLVITINGTGILIEIVFLTIFFVYCGRQKQRLIISAVIAAETAFIAILAVLVLTLQHTTEK
RTMSVGIVCCVFNVMMYASPLSVMKMVIKTKSVEFMPFWLSVAGFLNAGVWTIYALMPFD
PFMAIPNGIGCLFGLAQLILYGAYYKSTKRIMAERENQPGYVGLSSAIARTGSEKTANTNQE
PNNV

> AtSWEET8
MVDAKQVRFIIGVIGNVISFGLFAAPAKTFWRIFKKKSVEEFSYVPYVATVMNCMLWVFYGL
PVVHKDSILVSTINGVGLVIELFYVGVYLMYCGHKKNHRRNILGFLALEVILVVAIILITLFAL
KGDFVKQTFVGVICDVFNIAMYGAPSLAIIKVVKTKSVEYMPFLLSLVCFVNAGIWTTYSLIF
KIDYYVLASNGIGTFLALSQLIVYFMYYKSTPKEKTVKPSEVEISATERV

> AtSWEET9
MFLKVHEIAFLFGLLGNIVSFGVFLSPVPTFYGIYKKKSSKGFQSIPYICALASATLLLYYGIMK
THAYLIISINTFGCFIEISYLFLYILYAPREAKISTLKLIVICNIGGLGLLILLVNLLVPKQHRVSTV
GWVCAAYSLAVFASPLSVMRKVIKTKSVEYMPFLLSLSLTLNAVMWFFYGLLIKDKFIAMPN

ILGFLFGVAQMILYMMYQGSTKTDLPTENQLANKTDVNEVPIVAVELPDVGSDNVEGSVRPMK

> AtSWEET10
MAISQAVLATVFGILGNIISFFVCLAPIPTFVRIYKRKSSEGYQSIPYVISLFSAMLWMYYAMIKKDAMMLITINSFAFVVQIVYISLFFFYAPKKEKTLTVKFVLFVDVLGFGAIFVLTYFIIHANKRVQVLGYICMVFALSVFVAPLGIIRKVIKTKSAEFMPFGLSFFLTLSAVMWFFYGLLLKDMNIALPNVLGFIFGVLQMILFLIYKKPGTKVLEPPGIKLQDISEHVVDVVRLSTMVCNSQMRTLVPQDSADMEATIDIDEKIKGDIEKNKDEKEVFLISKN

> AtSWEET11
MSLFNTENTWAFVFGLLGNLISFAVFLSPVPTFYRIWKKKTTEGFQSIPYVVALFSATLWLYYATQKKDVFLLVTINAFGCFIETIYISMFLAYAPKPARMLTVKMLLLMNFGGFCAILLLCQFLVKGATRAKIIGGICVGFSVCVFAAPLSIIRTVIKTRSVEYMPFSLSLTLTISAVIWLLYGLALKDIYVAFPNVLGFALGALQMILYVVYKYCKTSPHLGEKEVEAAKLPEVSLDMLKLGTVSSPEPISVVRQANKCTCGNDRRAEIEDGQTPKHGKQSSSAAAT

> AtSWEET12
MALFDTHNTWAFVFGLLGNLISFAVFLSPVPTFYRICKKKTTEGFQSIPYVVALFSAMLWLYYATQKKDVFLLVTINSFGCFIETIYISIFVAFASKKARMLTVKLLLLMNFGGFCLILLLCQFLAKGTTRAKIIGGICVGFSVCVFAAPLSIIRTVIKTKSVEYMPFSLSLTLTISAVIWLLYGLALKDIYVAFPNVIGFVLGALQMILYVVYKYCKTPSDLVEKELEAAKLPEVSIDMVKLGTLTSPEPVAITVVRSVNTCNCNDRNAEIENGQGVRNSAATT

> AtSWEET13
MALTNNLWAFVFGILGNIISFVVFLAPVPTFVRICKKKSTEGFQSLPYVSALFSAMLWIYYAMQKDGTAFLLITINAFGCVIETIYIVLFVSYANKKTRISTLKVLGLLNFLGFAAIVLVCELLTKGSTREKVLGGICVGFSVSVFAAPLSIMRVVVRTRSVEFMPFSLSLFLTISAVTWLFYGLAIKDFYVALPNVLGAFLGAVQMILYIIFKYYKTPVAQKTDKSKDVSDHSIDIAKLTTVIPGAVLDSAVHQPPALHNVPETKIQLTEVKSQNMTDPKDQINKDVQKQSQV

> AtSWEET14
MVLTHNVLAVTFGVLGNIISFIVFLAPVPTFVRICKKKSIEGFESLPYVSALFSAMLWIYYALQKDGAGFLLITINAVGCFIETIYIILFITYANKKARISTLKVLGLLNFLGFAAIILVCELLTKGSNREKVLGGICVGFSVCVFAAPLSIMRVVIRTKSVEFMPFSLSLFLTISAITWLFYGLAIKDFYVALPNILGAFLGAVQMILYVIFKYYKTPLVVDETEKPKTVSDHSINMVKLSSTPASGDLTVQPQTNPDVSHPIKTHGGDLEDQMDKKMPN

> AtSWEET15
MGVMINHHFLAFIFGILGNVISFLVFLAPVPTFYRIYKRKSTESFQSLPYQVSLFSCMLWLYYALIKKDAFLLITINSFGCVVETLYIAMFFAYATREKRISAMKLFIAMNVAFFSLILMVTHFVVKTPPLQVSVLGWICVAISVSVFAAPLMIVARVIKTKSVEYMPFTLSFFLTISAVMWFAYGLFLNDICIAIPNVVGFVLGLLQMVLYLVYRNSNEKPEKINSSEQQLKSIVVMSPLGVSEVHPVVTESVDPLSEAVHHEDLSKVTKVEEPSIENGKCYVEATRPETV

> AtSWEET16
MADLSFYVGVIGNVISVLVFLSPVETFWRIVQRRSTEEYECFPYICTLMSSSLWTYYGIVTPGE
YLVSTVNGFGALAESIYVLIFLFFVPKSRFLKTVVVVLALNVCFPVIAIAGTRTLFGDANSRSS
SMGFICATLNIIMYGSPLSAIKTVVTTRSVQFMPFWLSFFLFLNGAIWGVYALLLHDMFLLVP
NGMGFFLGIMQLLIYAYYRNAEPIVEDEEGLIPNQPLLA

> AtSWEET17
MAEASFYIGVIGNVISVLVFLSPVETFWKIVKRRSTEEYKSLPYICTLLGSSLWTYYGIVTPGEY
LVSTVNGFGALVETIYVSLFLFYAPRHLKLKTVDVEAMLNVFFPIAAIVATRSAFEDEKMRSQ
SIGFISAGLNIIMYGSPLSAMKTVVTTKSVKYMPFWLSFFLFLNGAIWAVYALLQHDVFLLVP
NGVGFVFGTMQLILYGIYRNAKPVGLSNGLSEIAQDEEEGLTSRVEPLLS

> OsSWEET1a
MEHIARFFFGVSGNVIALFLFLSPVVTFWRIIKKRSTEDFSGVPYNMTLLNCLLSAWYGLPFV
SPNNILVTTINGTGSVIEAIYVVIFLIFAERKARLKMMGLLGLVTSIFTMVVLVSLLALHGQGR
KLFCGLAATIFSICMYASPLSIMRLVIKTKSVEFMPFLLSLSVFLCGTSWFIYGLLGRDPFIAIPN
GCGSFLGLMQLILYAIYRNHKGATPAAAAGKGDAADEVEDAKKAAAAVEMADAKTNKV
VADDADADADGKSADDKVASQV
> OsSWEET1b
MEDLAKFLFGVSGNVIALFLFLSPVPTFWRIIRRKSTEDFSGVPYNMTLINCLLSAWYGLPFV
SPNNILVSTINGAGAVIETAYVVVFLVFASTHKTRLRTLGLAAAVASVFAAVALVSLLALHGQ
HRKLLCGVAATVCSICMYASPLSIMRLVIKTKSVEYMPFLMSLAVFLCGTSWFIYGLLGRDPF
VTIPNGCGSFLGAVQLVLYAIYRNNKGAGGGSGGKQAGDDDVEMAEGRNNKVADGGAA
DDDSTAGGKAGTEV

> OsSWEET2a
MMNALGLSVAATSTGSPFHDVCCYGAGIAGNIFALVLFISPLPTFKRIVRNGSTEQFSAMPYI
YSLLNCLICLWYGLPFVSYGVVLVATVNSIGALFQLAYTATFIAFADAKNRVKVSSLLVMVFG
VFALIVYVSLALFDHQTRQLFVGYLSVASLIFMFASPLSIINLVIRTKSVEYMPFYLSLSMFLMS
VSFFAYGVLLHDFFIYIPNGIGTVLGVIQLVLYGYFRKGSREDSLPLLVTHT

> OsSWEET2b
MDSLYDISCFAAGLAGNIFALALFLSPVTTFKRILKAKSTERFDGLPYLFSLLNCLICLWYGLP
WVADGRLLVATVNGIGAVFQLAYICLFIFYADSRKTRMKIIGLLVLVVCGFALVSHASVFFFD
QPLRQQFVGAVSMASLISMFASPLAVMGVVIRSESVEFMPFYLSLSTFLMSASFALYGLLLRD
FFIYFPNGLGLILGAMQLALYAYYSRKWRGQDSSAPLLLA

> OsSWEET3a
MFPDIRFIVGIIGSVACMLLYSAPILTFKRVIKKASVEEFSCIPYILALFSCLTYSWYGFPVVSYG
WENMTVCSISSLGVLFEGTFISIYVWFAPRGKKKQVMLMASLILAVFCMTVFFSSFSIHNHHI
RKVFVGSVGLVSSISMYGSPLVAMKQVIRTKSVEFMPFYLSLFTLFTSLTWMAYGVIGRDPFIA
TPNCIGSIMGILQLVVYCIYSKCKEAPKVLHDIEQANVVKIPTSHVDTKGHNP

> OsSWEET3b
MVSNTIRVAVGILGNAASMLLYAAPILTFRRVIKKGSVEEFSCVPYILALFNCLLYTWYGLPV
VSSGWENSTVSSINGLGILLEIAFISIYTWFAPRERKKFVLRMVLPVLAFFALTAIFSSFLFHTH
GLRKVFVGSIGLVASISMYSSPMVAAKQVITTKSVEFMPFYLSLFSFLSSALWMIYGLLGKDLF
IASPNFIGCPMGILQLVLYCIYRKSHKEAEKLHDIDQENGLKVVTTHEKITGREPEAQRD

> OsSWEET4
MVSPDTIRTAIGVVGNGTALVLFLSPVPTFIRIWKKGSVEQYSAVPYVATLLNCMMWVLYGL
PAVHPHSMLVITINGTGMAIELTYIALFLAFSLGAVRRRVLLLLAAEVAFVAAVAALVLNLA
HTHERRSMIVGILCVLFGTGMYAAPLSVMKMVIQTKSVEYMPLFLSLASLVNGICWTAYALI
RFDLYITIPNGLGVMFAVAQLILYAIYYKSTQQIIEARKRKEADHVAMTDVVVDSAKNNPSS
GAAAAAANGRY

> OsSWEET5
MVMNPDAVRNVVGIIGNLISFGLFLSPLPTFVTIVKKKDVEEFVPDPYLATFLNCALWVFYG
LPFIHPNSILVVTINGTGLLIEIAYLAIYFAYAPKPKRCRMLGVLTVELVFLAAVAAGVLLGAH
TYDKRSLIVGTLCVFFGTLMYAAPLTIMKQVIATKSVEYMPFTLSLVSFINGICWTIYAFIRFDI
LITIPNGMGTLLGAAQLILYFCYYDGSTAKNKGALELPKDGDSSAV

> OsSWEET6a
MISPDAARNVVGIIGNVISFGLFLAPVPTFWRICKRKDVEEFKADPYLATLLNCMLWVFYGI
PVVHPNSILVVTINGIGLLVEGTYLLIFFLYSPNKKRLRMCAVLGVELVFMLAVILGVLLGAH
THEKRSMIVGILCVFFGSIMYFSPLTIMGKVIKTKSVEYMPFFLSLVCFLNGVCWTAYALIRFD
IYVTIPNGLGALFGAIQLILYACYYRTTPKKTKAAKDVEMPSVVVSGTGAAAAAGGGNTGG
GSVSVTVER

> OsSWEET6b
MISPDAARNVVGIIGNVISFGLFLSPVPTFWRICKRKDVEQFKADPYLATLLNCMLWVFYGI
PIVHPNSILVVTINGIGLIVEGTYLFIFFLYSPNKKRLRMLAVLGVELVFMLAVILGVLLSAHT
HKKRSMIVGILCVFFGSIMYFSPLTIMGKVIKTKSVEYMPFFLSLVCFLNGVCWTAYALIRFDI
YVTIPNGLGAIFGAIQLILYACYYRTTPKKTKAAKDVEMPSVISGPGAAATASGGSVVSVTVE
R

> OsSWEET7a
MVSPDMIRNVVGIVGNVISFGLFLSPVPTFWQIIKNKNKNKKKMEVVLAAEALFMAAVAL
GVLLGVHTHQRRSLIVGILCVIFDTIMYSSPLTVMSQVVKTKSVEYMPLLLSVVSFLNGLYWT
SYTLIRFDIFITIPNGLGVLFAAVQLILYVIYYRTTPKKQNKNLELPTVTPVAKDTSVGPISKDN
DLNGSTASHVTIDITIQP

> OsSWEET7b
MVSPDLIRNMVGIVGNIISFGLFLSPVPTFYRIIKNKDVQDFKADPYLATLLNCMLWVFYGL
PIVHPNSILVVTINGIGLIIEAVYLTIFFLFSDKKNKKKMGVVLATEALFMAAVVLGVLLGAH
THQRRSLIVGILCAIFGTIMYSSPLTIMSQVVKTKSVEYMPLLLSVVSFLNGLCWTSYALIRLDI
FITIPNGLGVLFALMQLILYAIYYRTTPKKQDKNLELPTVAPVAKDTSIVTPVSKDDDVVDGG

NASHVTINITIEP

> OsSWEET7c
MVSPDLIRNVVGIVGNVISFGLFLSPVPIFWRIIKNKNVQNFKADPILVVTINGISLVIEAVYLT
IFFLFSDKKNKKKMGVVLATEALFMAAVAVGVLLGAHTHQRRSLIVGILCVIFGTIMYSSPLT
IMVVKTKSVEYMPLLLSVVSFLNGLCWTLYALIRFDIFITIPNGLGVLFAIMQLILYAIYYRTTP
KKQDKNLELPTVAPIAKDTSIVAPVSNDDDVNGSTASHATINITIEP

> OsSWEET7d
MVPDLIRNVVGIVGNVISFGLFLSPVPTFWRIIKNKDVRDFKADQYLATLLNCMLWVFYGL
PIVHPNSILVVTINGIGLVIEAVYLTIFFLFSDKKNKKKMGVVLATEALFMAAVALGVLLDAH
THQRRSLIVGILCVIFGTIMYSSPLTIMSQVVKTKSVEYMPLLLSVVSFLNGLCWTSYALIRFDI
FITIPNGLGVLFALMQLILYAIYYRTTPKKPSTTGPHPRSRIRTSSYQPSPPSPRAPASSPLSART
TTSMAAMSPSISRLSHKLA

> OsSWEET7e
MVSPDLIRNVVGIVGNAISFGLFLSPVLTFWRIIKEKDMKYFKADPYLATLLNCMLWVFYGL
PIVHPNSILVVTINGIGLVIEAVYLTIFFLFSNKKNKKMGVVLATEALFMAAVALGVLLGAHT
HQRRSLIVGILCVIFGTIMYSSPLTIMSQVVKTKSVEYMPLLLSVVSFLNGLCWTSYALIRFDIF
ITIPNGLGVLFTLMQLILDKNQDKNLELPTVAPVAKETSIVTPVSKDDDINGSTASHVIINITK
EP
> OsSWEET11
MAGGSMANAVTSGVAGNSVAVATVYKKKSTGGYSSVYVVASSVWYAVKTNSRTNAGCGV
AAYVYVYARRARRTADVAAAVVTTYVKHVKGSVCASMAVVASKVKTKSVMGSVCTSAVA
WCYGTKDYVMYNVGGSCVMGYWYRKRNTAVTTSDSMSSAAAAATRVAGTHATSVSGVH
KVVVAAAADGVAAAAAADKNKVTAAV

> OsSWEET12
MVQALVFAVGIVGNILSFLVILAPVPTFYRVYKKKSTESFQSVPYAVALLSAMLWLYYALLTSD
LLLLSINSIGCLVESLYLTVYLLYAPRQAMAFTLKLVCAMNLALFAAVVAALQLLVKATDRR
VTLAGGIGASFALAVFVAPLTIIRQVIRTKSVEFMPFWLSFFLTLSAVVWFFYGLLMKDFFVAT
PNVLGLLFGLAQMVLYVVYKNPKKNSAVSEAAAAQQVEVKDQQQLQMQLQASPAVAPL
DVDADADADLEAAAPATPQRPADDDAIDHRSVVVDIPPPPQPPPALPAVEVA

> OsSWEET13
MAGLSLQHPWAFAFGLLGNLISFTTYLAPIPTFYRIYKSKSTEGFQSVPYVVALFSAMLWIFY
ALIKSNEALLITINAAGCVIETIYIVMYLAYAPKKAKVFTTKILLLLNVGVFGVILLLTLLLSHG
EQRVVSLGWVCVAFSVSVFVAPLSIIKRVIQSRSVEYMPFSLSLTLTLSAVVWFLYGLLIKDKYV
ALPNILGFTFGVVQMGLYVFYMNATPVAGEGKEGKGKLAAAEELPVVVNVGKLAAATPD
RSTGAVHVHPVPRSCAAEAAAAEPEVLVDIPPPPPPRAVEVAAV

> OsSWEET14
MAGMSHWAAGGNSMTYATYRYKSKSTGSVYVVASAMWYYAKSDCTNSAGCVTYAVYVY
AKKAKMTAKVNVGVGTSAGDRRVVGWVCVGSVSVVASRVVRTKSVMSSSTSAVVWYGK

DKYVANVGSGVMGYAMYRNSTKAVTKVAATATGDDDHSAAGVKHVVNAKSAAVDVVK
TRVHVDVSAADDKAAAATAAAVAGAGKKVAA

> OsSWEET15
MAFMSMERSTWAFTFGILGNLISLMVFLSPLPTFYRVYRKKSTEGFQSTPYVVTLFSCMLWM
YYAFVKSGAELLVTINGVGCVIETVYLAMYLAYAPKSARMLTAKMLLGLNIGLFGVIALVTL
LLSRGELRVHVLGWICVAVSLSVFAAPLSIIRLVIRTKSVEFMPFSLSFFLVLSAVIWFLYGLLKK
DVFVALPNVLGFVFGVAQMALYMAYRSKKPLVASSSSAVVAAGLEIKLPEHVKEVQAVAKG
AVAAAPEGRISCGAEVHPIDDVMPSEVVEVKVDDEETNRTDEMAGDGDHAMVRTEQIIKP
DMAIVVEV

> OsSWEET16
MADPSFFVGIVGNVISILVFASPIATFRRIVRSKSTEEFRWLPYVTTLLSTSLWTFYGLHKPGGL
LIVTVNGSGAALEAIYVTLYLAYAPRETKAKMVKVVLAVNVGALAAVVAVALVALHGGVR
LFVVGVLCAALTIGMYAAPMAAMRTVVKTRSVEYMPFSLSFFLFLNGGVWSVYSLLVKDYF
IGIPNAIGFALGTAQLALYMAYRRTKKPAGKGGDDDEDDEEAQGVARLMGHQVEMAQQR
RDQQLRKGLSLSLPKPAAPLHGGLDRIIKSFSTTPIELHSILHQHHGGHHHHHRFDTVPDD
DDEAVAAGGTTPATTAGPGDRH

>27613.m000628
MVIAKYSSNFVGIIGNTTSIGIFLLPAPTFYSMWKKQDIDQEFQFHPHLLKVQVCLLWIFYGL
PVVKPDRLLIATCNGLGLVVELVYLATFCFCDRENKGRTLVALGLAGEVIFTAVIVVVTLLDF
HTQDNRALLVGMFCVAFSVVMSSCGLGTMKKVIDTQDVESMPFNVSLANLANDCFWAAY
ALITTDHFVFFSYGIGALCSLAQLIVYACYYKPENDVLKLSKIHPSSG

>27985.m000892
MDVLHFLFGVFGNATALFLFLSPTITFKRIIKSKSTEQFSGIPYVMTLLNCLLSAWYGLPFVSK
NNLLVSTINGTGAVIETIYVLIFIIYAPRREKSKILGLFTLVLTIFALVAFVSLFALHGSTRKLFCG
LAATIFSIIMYASPLSIIRLVIKTKSVEFMPFFLSLFVFLCGTSWFIYGLLGRDPFVAIPNGFGCGL
GTLQLILYFIYRNSKASAEAKKQPTSQSMEMGPSGKPQKMVANGSQDEQV

>29475.m000237
MVSASAEFAHAVVGSIGNVISLILYLSPMPTFCHIYNQKDVEEFQCYPYVAAVMNCLLLIFQ
GLPMVAPSANSPFIFIINGLGLAVELLYLHIFRYYEKKHKGFSRVVLFLAAEVILLAIIVTAALL
GFHTHSNRNLFVGIFCAVSNVVMYGSPLAIMKKVVLTRSVEYMPHDLSLASFFNGVFWTVY
AVIIFDPLTLASNGLGALLSLAQLLLYAYYSNPKRTAAVMPVQLEPVI

>29579.m000197
MATLFSFSKSDIILTLGVLGNITTGLVYLAPVKTFWRIVVNKSTEEFESMPYICKLINAYCWVY
YGILKPNSILVATVNGFGAVCEIIFVLLFLLFAPPRMKFITAILAGVLDVGFPAAVVIITQLFLK
REAQIDVAGFFCVFFSMAAYGSPLSAMKTVITTKSVEFMPFLLSFFLFINGGVWTLYAILAKD
WFIGLPNGTGFGLGTAQMILYAIYYKRPQPQKHSDSLEDGWENECLISESDRITPKD

>29647.m002020

MALNDPRFILAFGILGNIVSFLVYLAPLPTFWRIVKKKSTEGFQSIPYSVALFSAMLTLYYATL
KENAILLITINSIGCLIEGIYLTIYMIYATQTSRVQIHFKLLILFNLGTYLLIVMLASELTHGTLRV
QVVGWICAVFSVCVFAAPLSIMRLVIKTKSVEYMPFSLSFFLTLCAISWLGYGLAVNDYFIASP
NILGFLFGIVQMVLYMIYKNKKNEILPTSTSQELAVSKPETSQDRENSNSSSLNQQDLEAAK
DDRRENNKAVPEEASERNGYRATCFGISF

>29726.m004066
MELSILFVGIIGNVISVLMFLSPVGTFWRIIKNESTEEFESLPYVCTLLNAALWTYYGIIKPGAY
LVATVNGFGIVVEIVYVALFLIYAPAKMRAKTAILVALLDVGFLAAAILVTRLALKGEVRIDA
TGFMCAGLNIIMYGSPLAAMKTVVTTKSVEFMPFFLSFFFFLNGGIWTFYAILTRDYFLGVP
NGTGFCLGITQLVLYAIYKNAKPCKTRVSDHRNGLEEGSQYENLISSSQTTPGN

>29822.m00334
MVNARTIVGIVGNIISFCLFLSPLPTFYRIIKKKDVEEFQFYPYVATVLNCMLWMFYGLPIVKE
DSLLVVTINSIGLVIELVYLGIYCFYDNQNKGRKKVGLCLLGEVGFMAVIIAIAMLAFHKLKY
RSLFVGVFCDILNVMMYSSPLLIMKKVIMTKSVEYMPFPLSLAGFLNGACWTAFAIIKLDLFI
LISNGLGTLAGAFQLIIFFRYYRWCAPKQTDDDDIVKPSEIQLSGANAASRV

>29822.m003349
MNCMLWNFYGLPMVHPGSTLLVTINSVGLALELIYITIFFIYAQRNGRLKVTGFLFMEFVVM
TALVSFTLKFYDNHGQRSTLVGIFCVVINILMYASPLTIMKKVIITKSVKYMPFCLSLATFLNG
AIVLYATVDIFDLFVLIASSVGVLSGVLQLILYACYYKAVPTLQVDDHHEKPADLQISVAVVD
EEKA

>29929.m004599
MAIISTHPPLAFAFGILGNIISILVYLAPVPTFYRIYRKKSTEGFQSLPYLVALFSSMLWLYYAML
KKDVFLLVTINAFGCVIETIYIIMYIIYATKKNRVSTFKVLTSMNLGLFAFIILFSHFLVKSSVRA
QVLGWICVAVSVCVFAAPLSIVAQVIKTRSVEFMPFNLSFFLTLSAIMWFAYGLSTKDTCVAL
PNVLGFILGLLQMVLYVIYRKAKKVILEEKLPEHLKTIVVLSTLGNSEQQLVTPVGSLLIFGQF
RDVATCRRFQILL

>30026.m001515
MPIVSPEVILVATVNSIGAIFQFIYILIFILHADKARKLKMIGLLVAVSALFAVIVFVSLNFFESH
ARQMFVGYLSVFSLISMFASPLCVINLVFKTKSVEYMPFYLSLATFLMSLSFFAYGMLKYDPFI
SVPNGIGTILGITQLMLYFYYSSKYGEGSRDPLLASYA

>30068.m002528
MVSSDTARNVVGILGNIISFFLFLSPVPTFIQIWKKRAVEQYSATPYLATLVNCMVWVLYGLP
MVHPNSLLVITINGTGTAIEILYLIIFIVYSDKKKRLKVVLAVLVEVIFVAVLALLVLTLAHTTK
KRSMIVGFVCICFNIMMYASPLSIMKMVITTKSVEYMPFFLSLASLANGVAWSSYAFIRFDPFI
FIPNGLGTLFALAQLALYAVFYKSTKRQIEARQGKAEVGLSEVVVNGNGNSKRTGPEPLNA
FGSEINGA

>30128.m008852

MANLSFIVGILGNIISILVFASPIKTFWIVMKKKSTENYKGVPYITTLLSTSLWTFYGLLNPDGL
LVVTVNGTGVVFQSVYVTLFLIYAPKDKKIKSAKLVALLNVGFVGAVIAVTLLAMHGHLRLT
FVGIVCAALTIGMYAAPLSAMRMVIKTKSVEYMPFLLSFFLFLNGGIWSIYALLVKDIYIGVP
NATGFVLGSVQLILYAIYKSKSPSTKPQDAIGEGSAHSVKGDIEMDAYSNDEEASAKNISLDK
GISLPVPSVNRQKSLQKVLRTLSLNAKDLQYWAIE

>30147.m013970
MDCRPTFRKIINQKAVEEFKPDPYLATVLNCAMWSFYGLPIVEEDSILVTTINAAGLVIELTY
VAIFFVFAPFHKRKKIVIVLVLELIIMAGVIIITMGIFSSIKKRATFVGILCIILNVIMYTSPLTVM
RMVIRTKSVKYMPFYLSLASLCNGLIWVAYAALRFDIYLVLPNGLGALSGLVQIVLYAIYYRT
TRWEDDDHETSRQPEVQVSSRV

>30147.m014444
MTMAEHHPLIFTFGVLGNIISILMFLSPMFTFIRVYKKKSTEGFQSIPYVVALFSCMLWIYYAM
LKSGDYLLLSINSFGCLVQTIYIVLFIFYAEKKAKILTLQLLFLMNFAGFLAIVALTRFFAKGSSR
LHIVGWFCVAVSAVLFAAPLSVIRLVVRTKSVEFMPFTLSLFLTLSAIMWLLYGVLLKDLYIAL
PNIFGLVFGAIQMVLYVIYRDGKKVIELPEKIDMDSPIKTFEVHAAVVSLPIPDDNYQVNKED
NPNEQRKPNADSTESLNQEQFTVDEVTA

>30147.m014445
MAIFNTHNPSVFVFGLLGNIVSFVVFLAPVPTFLRVCKKKSTEGFQSFPYVVSLFSAMLWLYY
ASLKSDAFLLITINSVGCLIETIYITLFITYAPKQARITTLKILLLLNFGGFCLILLLSHFLAKGSE
RATILGWVCVIFSVSVFAAPLSVMRIVIRTKSVEFMPFYLSFFLTLSAIMWLFYGLLLKDLYIAV
PNILGLVFGVLQMILYVIYKNVKTVVEEPKLPEHNVDNVKLSAVITCEVQQEVCSQSQPNG
DDGAHNKEQKMHDNPANAVTEYQRHSGMDASIADQSIACRV

>30147.m014446
MAYHLSLEFLFGVLANIISSMVCLAPLPTFYQICKKKTSEGFQSVPYVIALFSAMLWLFYATF
DDNATLLITINSFTFFMEVGYLSVYLFYGTRKDRMLTTKLVLFFNVFGFGMIAILTLFLTHGR
KRVDVLGWICMIFALCVFVAPLGIMRKVIKTKSVEFMPFSLSFFLTLSAVMWFFYGFLKKDIY
VYIPNVLGFFFGIVQMILYLIYRNSKKPVEEPKSQEFSEHIVDVAKLSAVICSELKTMVVAKLN
DNGNEVVKEETKNTKQEMEASNKV

>30147.m014447
MAFHLTLAFAFGLLGNIISFLVCLAPMPTFYQICKKKTSEGFQSIPYVIALFSATLWLFYAIFAN
DATLLITINSFAFFMETAYIAIYLFYAVKKDRLFTTKLVLSLNIFAFGSICVIAMFLTHGQKRVQ
LLGWICMVFALCVFVAPLAIVRKVIKTKSVEFMPFSLSFFLTLSAVMWFFYGFLKKDLYVAVP
NILGFMFGVLQMILYLIYRNPKKTGDDDQKANELPNQHSIIDVAKLNTRVSCCEPNATTVA
HSRNDREEQQTMQINREDKDATNTV

>30169.m006529
MGDRLRLAVGVMGNAASLLLYAAPILTFARVIRKRSIEEFSCVPYIVTLGNCLLYTWYGLPVV
SCRWENLPLVTINGLGIFFEISFILVYFRFAETRGKIKVAITIIPVILYFAATAAISSFAFHDHHHR
KLFTGSVGLLASVGMYGSPLVVMKQVITTKSVEFMPFYLSFFSFLASSLWLTYGLLSHDLFIAS

PNFLGVPFGIIQLVLYFIYRKWGVMEEPKDRDLERDNGEKSKQLKLAVDENTNGGKC
